# Improving school lunch menus with multi-objective optimisation: nutrition, cost, consumption and environmental impacts

**DOI:** 10.1017/S1368980023000927

**Published:** 2023-08

**Authors:** Alexandra L Stern, Stephen Levine, Scott A Richardson, Nicole Tichenor Blackstone, Christina Economos, Timothy S Griffin

**Affiliations:** 1 Tufts University Friedman School of Nutrition Science and Policy, 150 Harrison Ave, Boston, MA 02111, USA; 2 Tufts University College of Arts and Sciences, Medford, MA, USA; 3 Harvard University T.H. Chan School of Public Health, Boston, MA, USA

**Keywords:** School lunch, Menus, Climate change, Plate waste

## Abstract

**Objective:**

To support school foods programmes by evaluating the relationship between nutritional quality, cost, student consumption and the environmental impacts of menus.

**Design:**

Using linear programming and data from previously served menu items, the relationships between the nutritional quality, cost, student consumption and the environmental impacts of lunch menus were investigated. Optimised lunch menus with the maximum potential student consumption and nutritional quality and lowest costs and environmental impacts were developed and compared with previously served menus (baseline).

**Setting:**

Boston Public Schools (BPS), Boston Massachusetts, USA.

**Participants:**

Menu items served on the 2018–2019 BPS lunch menu (*n* 142).

**Results:**

Using single-objective models, trade-offs were observed between most interests, but the use of multi-objective models minimised these trade-offs. Compared with the current weekly menus offered, multi-objective models increased potential caloric intake by up to 27 % and Healthy Eating Index scores by up to 19 % and reduced costs and environmental impacts by up to 13 % and 71 %, respectively. Improvements were made by reducing the frequency of beef and cheese entrées and increasing the frequency of fish and legume entrées on weekly menus.

**Conclusions:**

This work can be extrapolated to monthly menus to provide further direction for school districts, and the methods can be employed with different recipes and constraints. Future research should test the implementation of optimised menus in schools and consider the broader implications of implementation.

The US National School Lunch Programme (NSLP) is a federally assisted meal programme which provides nutrition standards, monetary support and surplus foods to participating school districts. The programme acts as a vital safety net for low-income and food insecure children^(1)^. In 2019, the programme helped serve 4·8 billion lunches of which 74 % were free or reduced price for children^(2)^.

Schools participating in the NSLP operate with varied and sometimes competing interests; meals must be low cost, nutritious and acceptable to students. Additionally, some school districts face pressure to improve standards for sourcing and menu planning related to environmental sustainability, animal welfare and the local economy^(3,4)^. Balancing these demands can be challenging with limited resources.

Improving the nutritional quality of school meals remains a top priority because of the prevalence of childhood obesity and its association with an increased risk for early onset disease^(5,6)^. Nearly 20 % of children and adolescents in the USA are clinically obese, and this percentage is even greater for Hispanic and non-Hispanic black children^(7)^. To improve the nutritional quality and ensure nutritionally adequacy of school meals served through the US NSLP, the Healthy Hunger Free Kids Act of 2010 updated school meal nutrition standards by increasing requirements for fruits, vegetables and wholegrains and setting limits on calories and Na^(4)^. This policy undoubtedly improved the nutritional quality of school meals but raised concerns over the possibility of increased costs and student plate waste^(8–12)^.

Most school lunch revenue is generated from student lunch payments and monetary support from the federal government in the form of meal reimbursements^(13)^. For the average school food authority, the body which administers the NSLP in the USA, revenues do not outweigh costs; schools run a small deficit^(13)^. Fortunately, financial deficits have not been associated with the Healthy Hunger Free Kids Act and the resulting improvements in nutrition. Conversely, better nutritional quality has been shown to increase participation rates and result in greater revenue^(13–15)^. However, in some cases, better nutritional quality has been linked to greater food waste^(15)^.

Food waste is a pernicious problem, when students waste food they are not obtaining the full nutritional benefits of school meals. Additionally, when food is wasted, the limited resources embedded in the production of food such as time, money and the natural resources used to produce foods are also wasted^(16,17)^. Annual waste from school lunch has an estimated value of over one billion USD and, on average, about one-third of all milk and vegetables are wasted^(15,17)^. High waste rates might partially be explained by student dissatisfaction. In a nationally representative study, when students were asked if they liked school lunch, a majority of students (> 50 %) state that ‘school lunch is only okay’ and that they ‘sometimes’ like the taste of the food^(15)^. Striking balance across these interests can be challenging.

Added to these concerns is the growing pressure to incorporate environmental sustainability into menu planning^(18,19)^. The current food system uses large levels of natural resources (i.e. land and water) and contributes significantly to climate change and pollution^(20)^. Dietary choice influences the magnitude of these impacts. By serving lower environmental impact lunches to children, school districts can help students develop more environmentally sustainable eating habits for the future.

Research on European school districts has started to address school meal planning challenges through linear programming and optimisation. These studies focused primarily on cost and environmental outcomes, finding that menus defined using optimisation could reduce greenhouse gas emissions by up to 60 % while meeting cost standards^(21–23)^. Additionally, in a Swedish school district, when optimised menus were served, there was no difference in student satisfaction, consumption or waste^(24,25)^. Using similar methods, US school districts could gain insight into balancing improved nutritional quality, student acceptance and reduced costs while managing the environmental impacts of their menus.

Given the obstacles that school districts face in harmonising nutritional quality, cost, consumption and environmental sustainability, the objectives of this research were to investigate how these variables interact when designing elementary school menus. Through a collaboration with a large urban school district in the Northeastern US, this modelling study used integer linear programming and integer goal programming to develop weekly school lunch menus with varying goals. We then compared the modelled menus with current menus offered (baseline) to make recommendations for improving school lunch menu planning.

## Methods

### Data

#### Menu, nutrition and cost

Boston Public Schools (BPS) is a large urban school district in the USA which serves over 50 000 students daily and participates in the NSLP. The school district provided data on the cost and nutrient content of menu items offered to kindergarten through fifth grade students during the 2018–2019 school year. Where nutrition data were missing, the Food and Nutrient Database for Dietary Studies was used. In total, the school district provided data on 142 menu items: seventy-seven entrées, eighteen fruit sides, forty-three vegetables sides and four milk options. For modelling purposes, entrées were divided into two subcategories (subcategory 1, entrée protein types; subcategory 2, flavour profiles) to limit the repetition of protein types and provide unique flavours when developing optimised menus. Details of the menu items including their names, cost, nutrient content and baseline frequency offered are provided in online supplementary material, Supplemental Table 1.

Lunches served by BPS comply with the NSLP nutrition standards. Under these standards, lunches are required to contain five meal components (meat or meat alternative, grain, vegetable, fruit and milk)^(26)^. For the purposes of these analyses, we refer to the combination of meat or meat alternative and grains as an entrée. Additionally, the NSLP nutrition standards have daily or weekly requirements for fruit, vegetables, vegetable subgroups, grains, meats or meat alternatives and fluid milk, with limits to calories, Na and saturated fat. Meeting these standards ensures that children receive a nutritionally adequate lunch. Further details on these standards are outlined elsewhere^(27)^.

BPS provides lunch to all students for free regardless of economic status. As such, during the 2018–2019 school year BPS received the federal free lunch reimbursement rate of 3·33 USD per lunch^(28)^. Of this, the school district’s food budget was 1·34 USD per lunch.

The remaining funds were used for staff and other operations costs.

#### Baseline menus offered

The baseline menus offered during the 2018–2019 school year were used as a comparison for menus developed using optimisation. We calculated the average baseline weekly cost, nutritional quality, consumption rates and environmental impacts of menus offered using the 2018–2019 school year menus and purchasing records. Purchasing records were used specifically for milk and fruit, because details on the types of these items were not listed on menus.

#### Participation and consumption

Participation is the rate at which students purchase lunch from the school. In BPS, participation was consistent across menus and was therefore not incorporated into the model.

Greater consumption means less waste, more efficient use of school resources and allows students to receive the full nutritional benefit of the lunch. To develop school lunch menus with greater *potential* consumption and understand how consumption might change under different menu planning goals, real data on student consumption were used to create modelled menus.

The average grams consumed of each menu item were collected from 7000 lunches served across 50 d in twelve schools during the 2018–2019 school year from baseline menus offered. This research used standard plate waste techniques for quantifying student consumption which are described elsewhere^(29)^. Briefly, we measured the starting weight of foods served in grams, collected trays when students finished their lunches and weighed any remaining food. Consumption was calculated as the difference between the starting weight of food served and the weight of remaining food. Consumption calculations only accounted for edible portions of foods (i.e. did not include banana peels, food packaging, etc.).

As a modelling study, when describing modelled or optimised menus, we use the term consumption to mean potential or theoretical consumption, because optimised menus were not served to students. We present results as offered and potentially consumed and explored the relationship between these variables. Specifically, we explore the relationship between offered and consumed for energy, nutritional quality and cost. With this comparison, we examined the efficiency of the district’s budget (i.e. spending 1·34 USD per lunch but only 0·5 USD is consumed), and if offering menus with high nutritional quality led to improvements in dietary quality.

#### Global warming potential and water scarcity

To quantify the environmental impacts of menus, we included data on the global warming potential (GWP) and water scarcity associated with each menu item. These impact categories are major areas of environmental concern and previous research suggests that land use and marine eutrophication potential are strongly and positively correlated with GWP^(27)^. Data were available from Stern *et al*. and Heller *et al*.^(30,31)^.

### The Healthy Eating Index

We used the Healthy Eating Index (HEI) to assess the nutritional quality of baseline and optimised weekly menus. The HEI measures the proximity of a diet or menu to the recommendations in the US Dietary Guidelines for Americans. The index includes thirteen dietary components divided into categories to encourage or discourage (Table 1). Greater quantities of components to encourage and smaller quantities of components to discourage yield higher scores. Each component is scored separately, and the final score is the sum of the component scores. The maximum score, representing the greatest nutrition quality, is 100. Units for the meal components (cup eq. and oz. eq.) are from the US Dietary Guidelines for Americans and compensate for the different densities of foods^(32)^.

**Table 1:** Components and scoring for the Healthy Eating Index 2015*

Component	Maximum points		Standard for minimum score
Adequacy			
Total fruit†	5	≥ 0·8 cup eq. per 1000 kcal	No fruit
Whole fruit‡	5	≥ 0·4 cup eq. per 1000 kcal	No whole fruit
Total vegetable§	5	≥ 1·1 cup eq. per 1000 kcal	No vegetables
Greens and beans§	5	≥ 0·2 cup eq. per 1000 kcal	No dark-green vegetables or Legumes
Wholegrains	10	≥ 1·5 oz. eq. per 1000 kcal	No wholegrains
Dairy¶	10	≥ 1·3 cup eq. per 1000 kcal	No dairy
Total protein foods§	5	≥ 2·5 oz. eq. per 1000 kcal	No protein foods
Seafood and plant proteins§,||	5	≥ 0·8 oz. eq. per 1000 kcal	No seafood or plant proteins
Fatty acids**	10	(PUFA + MUFA)/SFA ≥ 2·5	(PUFA + MUFA)/SFA ≤ 1·2
Moderation			
Refined grains	10	≤ 1·8 oz. eq. per 1000 kcal	≥ 4 oz. eq. per 1000 kcal
Na	10	≤ 1·1 grams per 1000 kcal	≥ 2 g per 1000 kcal
Added sugars	10	≤ 6·5 % of energy	≥ 26 % of energy
Saturated fats	10	≤ 8 % of energy	≥ 16 % of energy

* Intakes between the minimum and maximum standards are scored proportionately.

† Includes 100 % fruit juice.

‡ Includes all forms except juice.

§ Includes legumes (beans and peas).

|| Includes seafood, nuts, seeds, soya products (other than beverages) and legumes (beans and peas).

¶ Includes all milk products, such as fluid milk, yogurt and cheese, and fortified soya beverages.

** Ratio of PUFA and MUFA to SFAs.

### Linear programing

Linear programming and optimisation are tools to find the best solution among many feasible solutions. When planning school lunches, there are numerous possible menu combinations which satisfy the NSLP requirements for nutritional adequacy and cost requirements for the district. Optimisation considers the possible combinations and finds the solution which meets a particular goal. In this case, the weekly menu with the greatest nutritional quality and consumption rates and the lowest costs and environmental impacts.

Each linear programming or optimisation model includes three components: *decision variables*, *objective functions* and *constraints*. In this study, the *decision variables* were the number of servings of each menu item selected for a weekly menu. The *objective functions* maximised consumption and nutritional quality and minimised cost and environmental impacts, and the *constraints* reduced repetitions of similar foods and were the nutrition standards which ensure nutritionally adequacy of meals and reimbursement rates (USD) for the NSLP. A week of lunch (5 d) was chosen as the unit of analysis because the US NSLP guidelines are based on weekly standards and school lunch programmes typically follow 3–5-week menu cycles that repeat across the school year. To consider trade-offs across the goals, we used both single- and multi-objective models which are further explained in the objective function section.

We used two forms of linear programming, integer linear programming and integer goal programming, and as such all objective functions and constraints were linear. Throughout the paper, we refer to these methods as optimisation.

#### Constraints

All decision variables were subject to constraints to ensure that optimised menus were nutritionally adequate, cost less than or equal to the federal reimbursement rate for food and provided adequate variety across the week (Table 2). All modelled menus were constrained by the nutrition standards for the NSLP and were thus nutritionally adequate^(27)^. Variety is important for nutrition and student acceptance; therefore, we included constraints on the number of times a food could be served within the week. Additionally, an integer constraint was applied to the decision variables to ensure that partial portions of foods were not selected.

**Table 2:** Nutrition, cost and repetition constraints for school lunch models

Constraints		Lower bound	Upper bound
Nutrition/meal pattern			
Energy (kcal)		≥ 2750	≤ 3250
Saturated fat (g)			≤ 10 % of kcal
Na (mg)			≤ 1230 on average lunch^−1^
Fruit (cup eq.)		≥ 2·5	
Vegetable (cup eq.)		≥ 3·75	
	Red orange	≥ 0·75	
	Dark green	≥ 0·5	
	Starchy	≥ 0·5	
	Other	≥ 0·5	
	Beans and peas	≥ 0·5	
Grain (oz. eq.)		≥ 8	≤ 9
Wholegrain (oz. eq.)		≥ ½ Grains*	
Meat/meat alternative (oz. eq)		≥ 8	≤ 10
Milk (cups)		≥ 5	
Repetition constraints			
Entrées			≤ 1
Fruits and vegetables			≤ 2
Entrée protein types			
	Beef		≤ 2
	Chicken		≤ 2
	Turkey		≤ 2
	Vegetarian		≤ 2
	Fish		≤ 1
Entrée flavour profiles			
	Soy/orange		≤ 1
	Barbecue		≤ 1
	Tomato salsa		≤ 1
	All Spice/turmeric		≤ 1
	Salad		≤ 1
	Pasta		≤ 1
	Tofu		≤ 1
	Cheese		≤ 1
Total weekly components			
	Entrée		= 5
	Fruit		= 5
	Vegetable		= 10†
	Milk		= 5
Cost (USD)			≤ 6·7

* All grains served in Boston Public Schools are whole grain or whole grain rich.

† The school district offers up to two vegetables each day to meet vegetable subgroup requirements.

#### Objective functions

We used seven objective functions to compare menus with single and/or multiple goals. The objective functions can be divided by their goals: maximise consumption (O1-O2), minimise cost (O3), maximise nutritional quality (O4) or a combination of goals, which we refer to as multi-objective functions (O5-O7).

Objective functions O1 and O2 developed weekly menus with the greatest potential consumption by maximising the total energy (kcal) consumed (O1) and total value (USD) consumed (O2). We maximised the value consumed to understand how efficient the district was with its budget. Objective functions O5, O6 and O7 used goal programming to simultaneously achieve consumption, cost, nutritional quality and environmental goals. These objective functions are referred to as the *multi-objective functions* because they simultaneously consider multiple disparate goals. To simultaneously achieve these goals, these objective functions minimised the sum of the relative deviations from each goal. Objective functions O5, O6 and O7 were identical except in the weighting of goals. In objective function O5, equal weights were applied to each goal; in objective function O6 we applied a weight of 10 to the calories consumed goal. In objective function O7, we applied a weight of 30 to the calories consumed goal, because increasing consumption is the primary goal of the district and reducing environmental impacts has been associated with improved nutritional quality^(33)^.

More detailed descriptions of each of the objective functions including the mathematical expressions are provided in the supplemental information.

#### Models

Optimisation models combine constraints and objective functions. Here, we use seven models to create and compare seven unique menus. All models were run in Microsoft Excel for Microsoft 365 MSO 16.0 in Solver with the Simplex algorithm for linear models. Table 3 lists the acronyms associated with each model used in the results. We refer to models which maximised consumption and nutritional quality while minimising cost and environmental impacts simultaneously (O5-O7) as multi-objective models or menus, and other models are referred to as single-objective models.

**Table 3:** Optimisation models for balancing cost, nutritional quality, consumption and environmental impacts

Objective function		Acronym
Single-objective models (menus)		
O1	Maximise energy (kcal) consumed	MAX-CALCON
O2	Maximise value (USD) consumed	MAX-COSTCON
O3	Minimise cost (USD)	MIN-COST
O4	Maximise nutritional quality (HEI components)	MAX-HEI
Multi-objective models (menus)		
O5	Minimise deviations of goals (energy consumed, cost, HEI and environmental impacts)	MIN-DEV
O6	Minimise deviations of goals (energy consumed, cost, HEI and environmental impacts) with consumption weights × 10	MIN-DEV10
O7	Minimise deviations of goals (energy consumed, cost, HEI and environmental impacts) with consumption weights × 30	MIN-DEV30

## Results

### Baseline menus offered

**Fig. 1:**
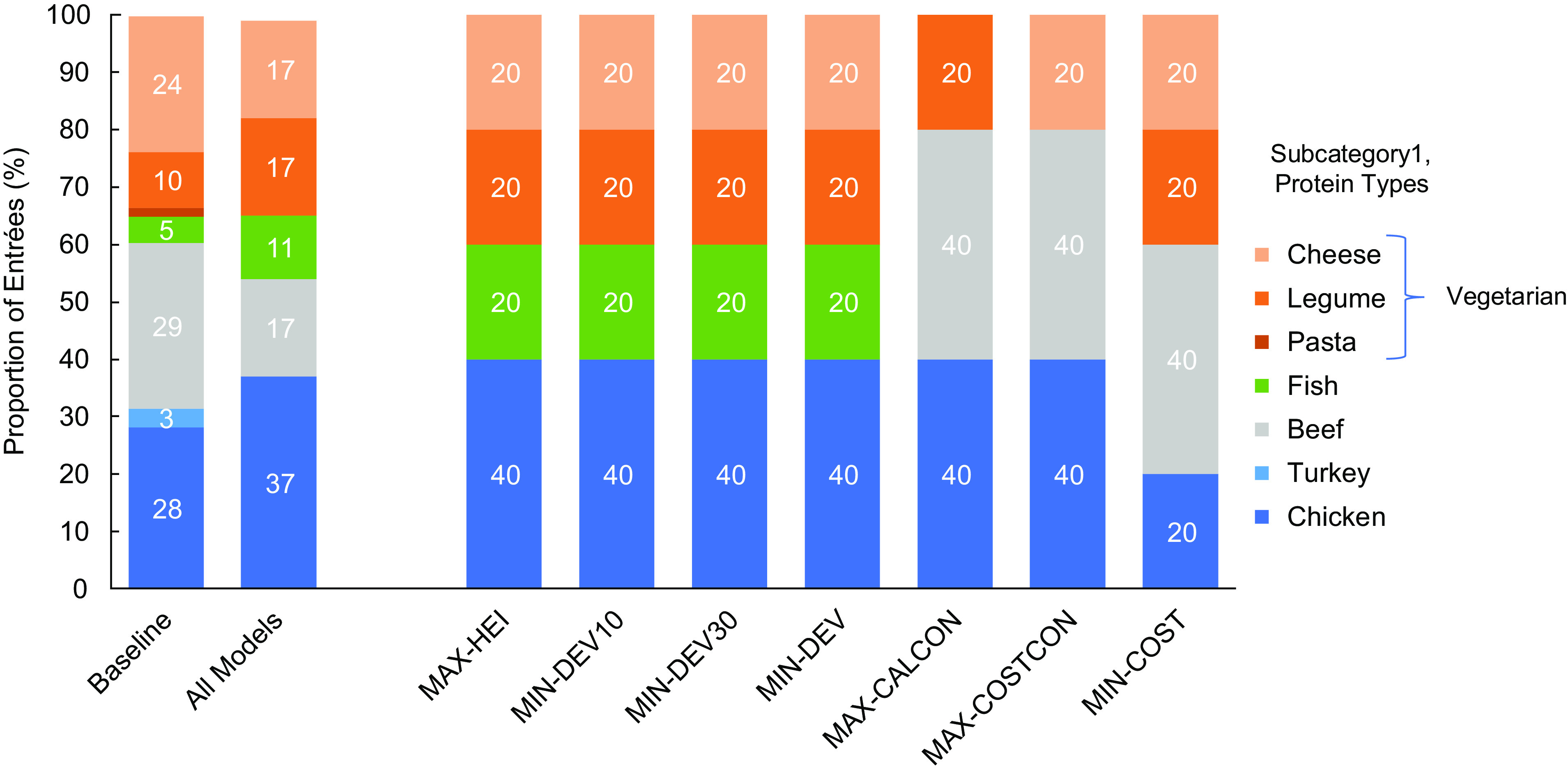
Composition of optimised and baseline menus by entrée protein type. The proportion of each entrée protein type was determined for baseline and optimised weekly menus and all optimised weekly menus combined (all models). Each bar represents the entrées selected by the models or at baseline. Each optimised menu included five entrées, all models combined represents thirty-five entrées, and the baseline column includes 800 entrées. Different colours distinguish the protein types, with vegetarian proteins in shades of orange and poultry in shades of blue. The proportion of each entrée protein type was determined for baseline and optimised weekly menus and all optimised weekly menus combined (all models). Each bar represents the entrées selected by the models or at baseline. Each optimised menu included five entrées, all models combined represents thirty-five entrées, and the baseline column includes 800 entrées

**Fig. 2:**
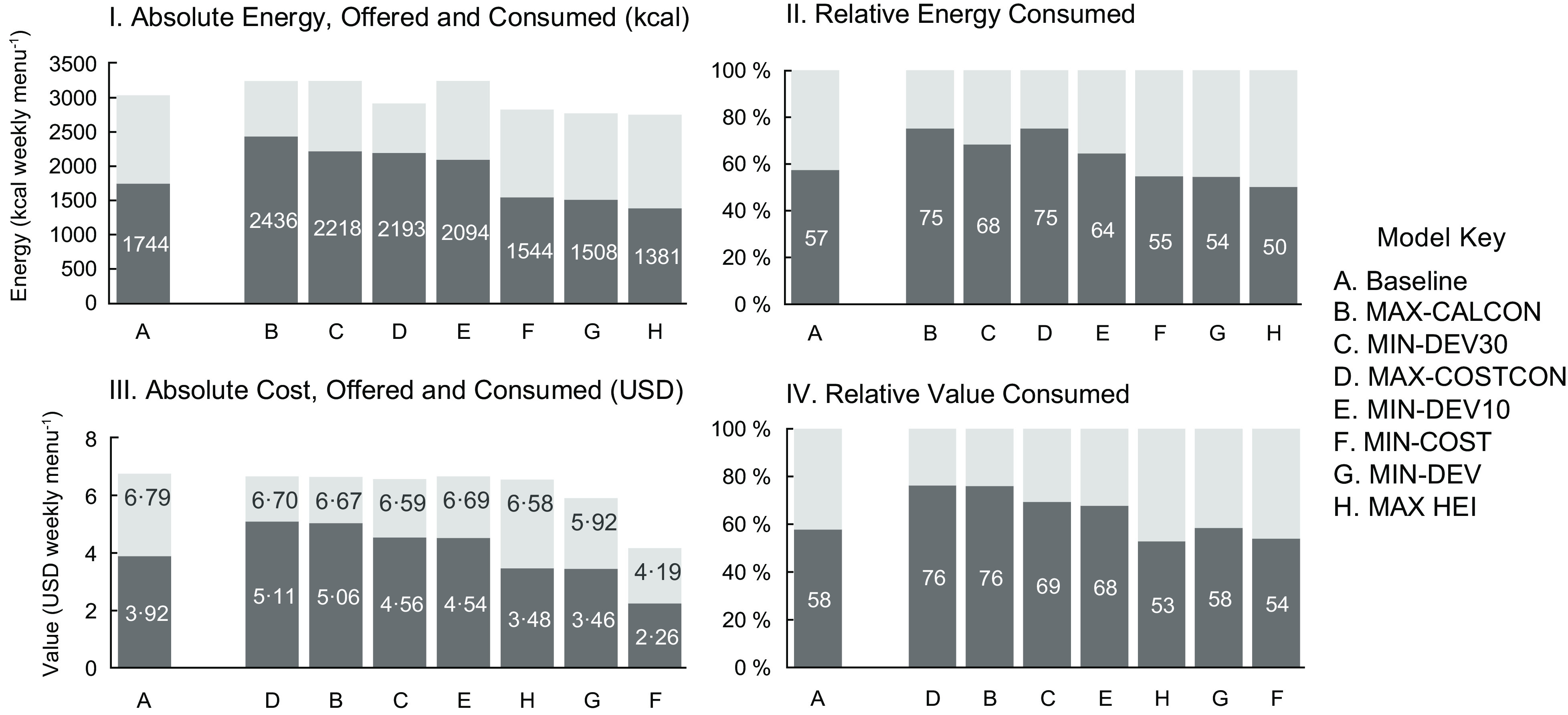
Energy (kcal) and cost (USD) offered and consumed of baseline and optimised weekly menus. Energy and cost, offered and consumed, were determined for optimised and baseline weekly menus. Figures are divided into absolute and relative results for energy and cost/value: I. Absolute energy, offered and consumed (kcal), II. Relative energy consumed, III. Absolute cost, offered and consumed (USD) and IV. Relative value consumed. Full columns (including dark and light shading) represent energy and cost of weekly menus offered and darker shading represents the proportion of energy or value consumed. Values in white inside of columns are the absolute and relative energy and value consumed. Columns are ordered from greatest to least absolute energy or cost consumed, and corresponding relative graphs follow this order

**Fig. 3:**
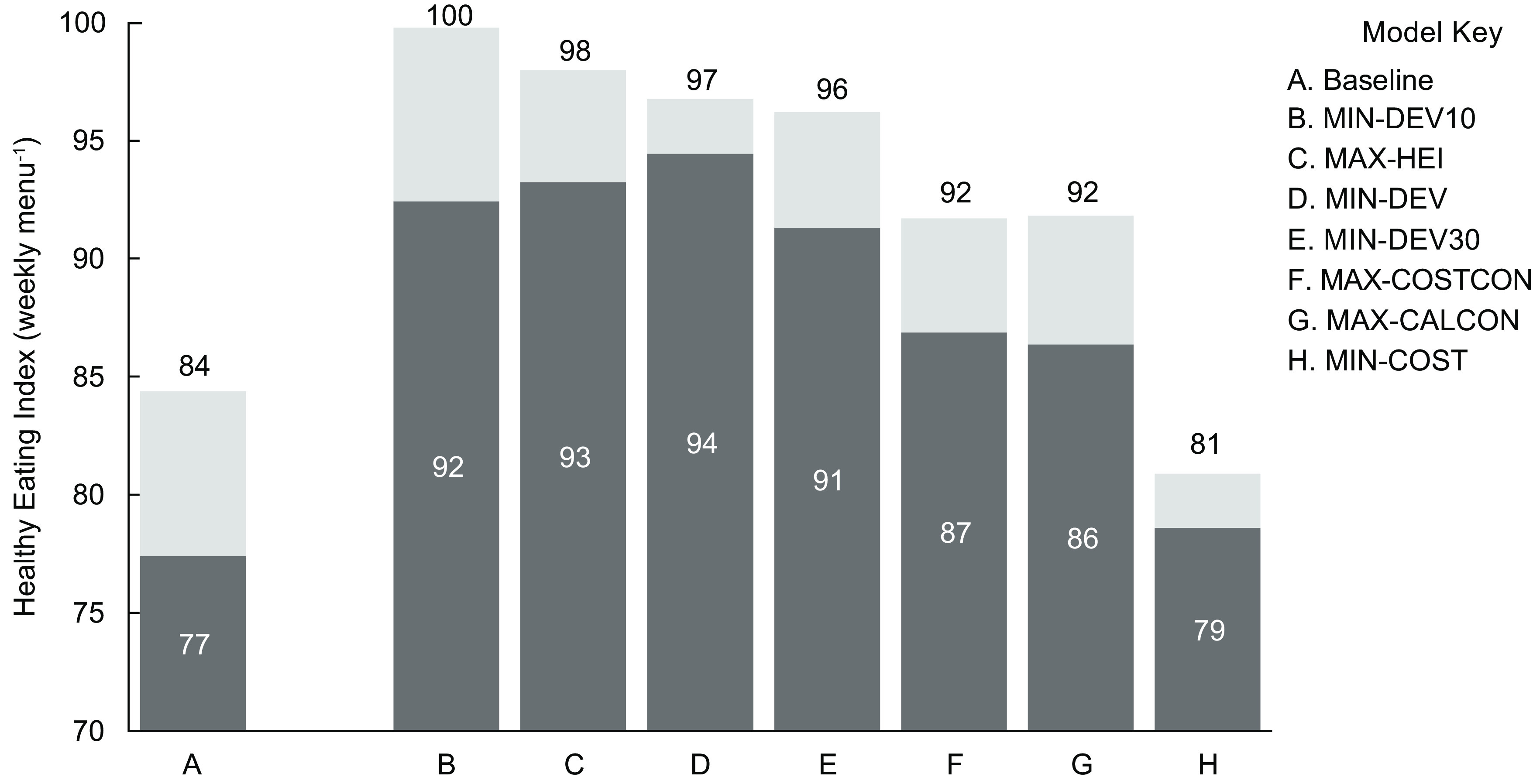
Healthy Eating Index (HEI) of baseline and optimised weekly menus, as offered and consumed. Columns represent the HEI of baseline and optimised weekly menus, with darker shaded portions of columns representing the HEI of the consumed portion. Labels for the HEI of the consumed portion are in white and labels for the HEI offered are in black. The y-axis starts at 70 to emphasise differences

**Fig. 4:**
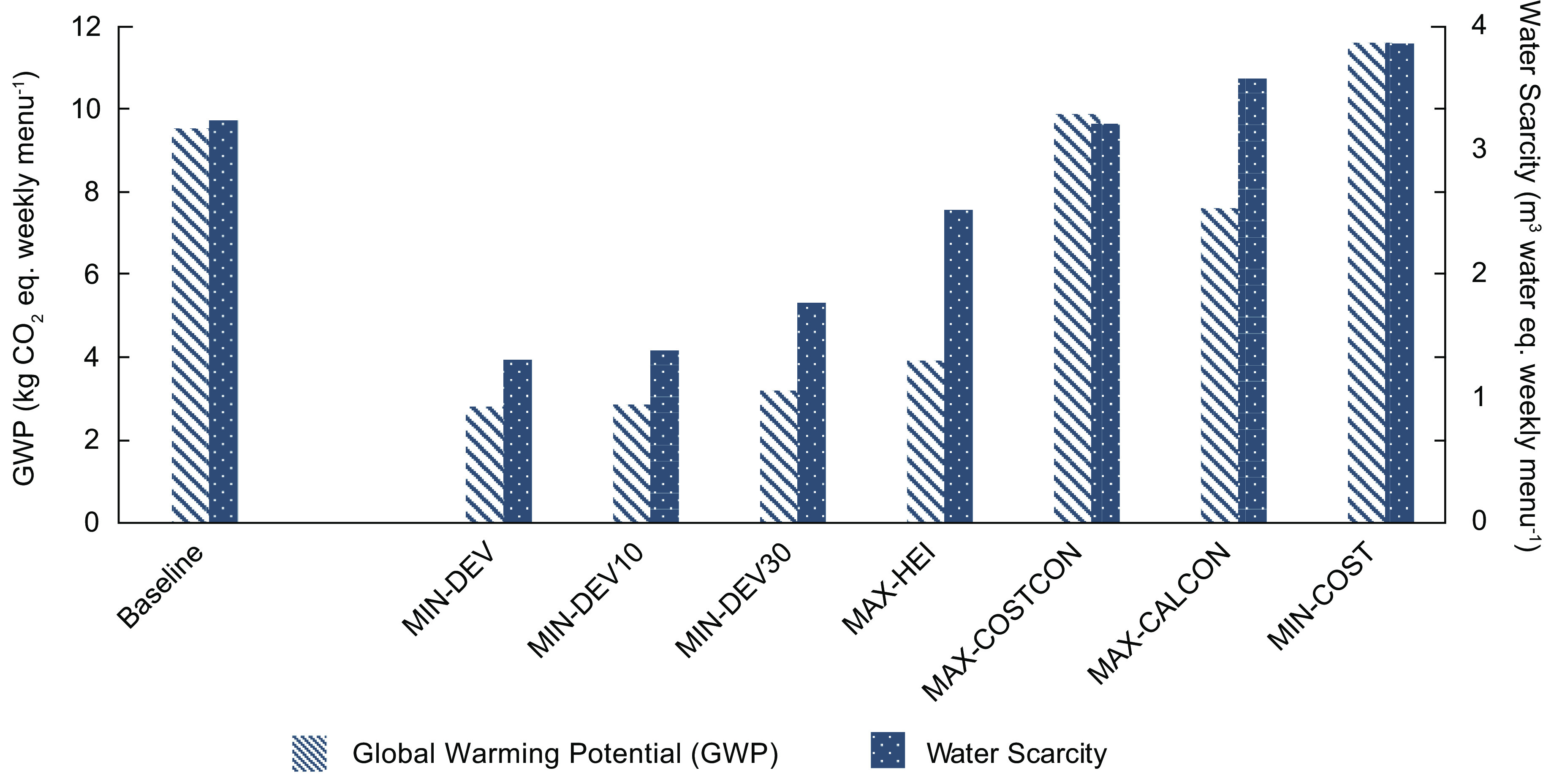
Global warming potential (GWP) and water scarcity of baseline and optimised weekly menus. Columns with stripes represent the GWP (kg CO_2_ eq.), and columns with dots represent water scarcity (m^3^ water eq.) of baseline and optimised weekly menus

Baseline menus offered mostly beef (29 %), chicken (28 %) and vegetarian (34 %) entrées, with fewer entrées containing turkey (3 %) and fish (5 %) (Fig. 1). Table 4 lists the average consumption rates and costs by entrée protein type, as well as the entrees with the greatest potential proportion consumed and the lowest cost per serving. There was no overlap between the ten entrées with the greatest consumption and the ten least cost entrées. On average, beef entrées had the greatest proportion consumed, followed by cheese. The least cost entrées contained legumes, followed by beef. On average, students consumed 1744 kcal or 3·92 USD of lunches offered (Fig. 2). Baseline weekly menu costs (6·79 USD) were slightly above the allocated funds available (Fig. 2). Like most other school districts in the USA, this suggests that the district runs a small deficit. The average HEI of baseline menus was 84 (Fig. 3) and the GWP and water scarcity were 9·5 kg CO_2_ eq. and 3·2 m^3^ water eq., respectively (Fig. 4).

**Table 4:** Average consumption rates and cost by entrée protein type and entrées with the greatest consumption (%) and lowest costs (USD)

Entrée protein/entrée	Avg. consumption (%)*	Cost (USD serving^−1^)
Beef	68	0·66
Chicken	57	0·88
Fish	59	0·91
Turkey	50	1·10
Vegetarian	52	0·71
Cheese	67	0·75
Pasta	40	1·36
Legume	43	0·56
Greatest consumption (descending order)		
Hot dog on a bun with popcorn	88	0·99
Meatball pizza	87	0·74
Veggie pizza	85	0·74
Cheese pizza	84	0·74
White garlic pizza	84	0·74
Chicken tenders with cheese bites	79	1·20
Pulled BBQ chicken with cornbread	78	0·80
Pulled BBQ chicken sandwich	78	0·55
Cheese bites	77	0·74
Meatballs and garlic bread	76	0·52
Least cost (ascending order)		
Marinated tofu with brown rice	40	0·28
Hamburger	71	0·29
Marinated tofu with Lo Mein noodles	40	0·29
Cheeseburger	71	0·34
Grilled cheese	57	0·37
Three bean chili with tortilla chips	60	0·38
Seasoned chicken and brown rice	46	0·40
Marinated tofu with vegetable fried rice	40	0·41
Toasted cheese sandwich and three bean chili	53	0·45
Macaroni and cheese	62	0·46

* Consumption was calculated as the average grams consumed divided by the average starting weight.

### Composition

All optimised menus contained chicken and vegetarian entrées (Fig. 1). Beef entrées were only selected by the models which minimised cost (MIN-COST) and maximised consumption (MAX-CALCON, MAX-COSTCON). The models which maximised nutritional quality (MAX-HEI) and were multi-objective (MIN-DEV, MIN-DEV10, MIN-DEV30) included fish entrées. Overall, optimisation models selected about double the proportion of legume and fish entrées and about half the proportion of cheese and beef entrées as baseline menus.

While all models selected vegetarian entrées, the types of vegetarian entrées differed across models (online supplementary material, Supplemental Table 3). The models which maximised consumption (MAX-CALCON, MAX-COSTCON) selected pizza and peanut butter and jelly sandwiches, whereas marinated tofu entrées were selected by the models which maximised nutritional quality (MAX-HEI) and were multi-objective (MIN-DEV, MIN-DEV10). The MIN-DEV30 model, which weighted consumption the greatest, included both pizza and marinated tofu with brown rice.

Fresh bananas were selected for nearly all optimised menus and fresh watermelon was selected by models which maximised consumption (MAX-CALCON, MAX-COSTCON) and minimised cost (MIN-COST). Compared with baseline, the prevalence of bananas and watermelon on optimised menus was up to four times greater, but the prevalence of fresh apples was up to four times less (online supplementary material, Supplemental Figure 1). The most selected vegetables for optimised menus included cherry tomatoes, raw romaine lettuce, roasted red potatoes and plantain slices (online supplementary material, Supplemental Figure 2). Plantain slices were selected by models which maximised consumption (MAX-CALCON, MAX-COSTCON) and multi-objective models with greater consumption weighting (MIN-DEV10, MIN-DEV30). Compared with baseline menus offered, optimised menus selected cherry tomatoes more often than sweet potato wedges, which fulfilled the vegetable subgroup requirement for red/orange vegetables. Another major difference between baseline and optimised menus was the prevalence of sweet corn, which was on optimised menus nearly four times less than baseline menus.

### Consumption and cost

Menus which maximised consumption (MAX-CALCON, MAX-COSTCON) increased potential student consumption by up to 692 kcal and 1·15 USD above baseline weekly menus (Fig. 2). These increases are approximately equivalent to students consuming an extra carton of milk each day. The proportion consumed relative to the energy or value offered was also greatest for these models (75 % for energy and 76 % for value).

Weekly menus that minimised costs (MIN-COST) and maximised nutritional quality (MAX-HEI) had lower energy (kcal) and value (USD) consumed than baseline menus offered in absolute and relative terms (Fig. 2). This suggests a potential trade-off between maximising consumption and nutrition and cost goals.

The least cost menu (MIN-COST) and multi-objective menu with equal weights (MIN-DEV) reduced the cost of meals by 2·60 and 0·87 USD, respectively. Extrapolated over a year, the least cost menu would provide potential savings of 93·6 USD per student or about 5M USD for the whole of BPS. Due to the cost constraint, all other optimisation models produced weekly menus which cost less than or equal to the cost constraint of 6·70 USD per weekly menu (or 1·34 USD per lunch).

### Nutritional quality

Compared with baseline menus, optimised menus which maximised nutritional quality (MAX-HEI) and were multi-objective (MIN-DEV, MIN-DEV10, MIN-DEV30) had 19–32 % greater fibre and less added sugar (89–94 %), saturated fat (51–77 %) and Na (3–26 %) (online supplementary material, Supplemental Table 4). These optimised menus also had greater servings of total vegetables, beans and peas, dark green vegetables, plant proteins and seafood and offered more than the daily Dietary Reference Intake for Ca, fibre and Fe^(34)^. The HEI of these menus was up to 19 % greater than baseline menus offered or about 16 points higher (Fig. 3). Contrary to what would be expected, these optimised menus had lower total dairy and Ca than baseline menus, but this did not impact HEI because the maximum component score for dairy was met.

Menus which maximised consumption (MAX-CALCON, MAX-COSTCON) also had greater HEI than baseline menus because they contained less Na and saturated fat per 1000 kcal (Fig. 3). These differences added about eight additional points to the HEI of these menus. While there appeared to be a trade-off between maximising consumption and nutritional quality, this result suggests that nutrition and consumption might be more aligned than previously expected. The model which minimised cost, however, had the lowest HEI and was three points below baseline offerings, highlighting that nutritional quality and cost minimisation are possibly at odds.

The potential HEI consumed was calculated using the components consumed and energy offered. Although the MAX-HEI and MIN-DEV10 models had the greatest HEI offered, the MIN-DEV model had a slightly greater HEI consumed (Fig. 3). In most cases, the models which maximised consumption and were multi-objective with greater consumption weighting (MAX-CALCON, MAX-COSTCON, MIN-DEV10, MIN-DEV30) resulted in greater absolute and relative consumption of important nutrients such as fibre, Ca and Fe, compared with the MAX-HEI model (online supplementary material, Supplemental Table 4). For example, the MIN-DEV10 menu had the highest potential fibre consumption (32 g) but offered less fibre than the model which maximised HEI. All multi-objective models were associated with potential consumption of more than 100 % of daily fibre and Fe and 90 % of daily Ca Dietary Reference Intakes^(34)^.

### Environmental impacts

Menus which maximised nutritional quality (MAX-HEI) and were multi-objective (MIN-DEV, MIN-DEV10, MIN-DEV30) reduced GWP and water scarcity by on average 67 % and 46 % compared with baseline, respectively (Fig. 4). The multi-objective weekly menu with equal weights (MIN-DEV) was the most effective at reducing GWP and water scarcity and had the greatest HEI consumed of all models, suggesting synergy between reducing environmental impacts and maximising nutritional quality. In most cases, the other models maintained or increased environmental impacts above baseline menus offered, suggesting that reducing costs and increasing consumption in this context do not necessarily promote environmental sustainability.

## Discussion

This research designed elementary school lunch menus with improved nutritional quality, increased potential consumption rates, lower costs and reduced environmental impacts using linear programming and optimisation. Menus which maximised nutritional quality had slightly lower potential consumption of important nutrients and HEI consumed compared with menus which also maximised consumption. As such, incorporating consumption rates into optimisation models could be essential to ensuring that improved nutrition is realised. Trade-offs between cost, consumption and nutrition were apparent when modelling menus with single objectives. Multi-objective menus minimised these trade-offs and produced improvements across all goals compared with baseline. School districts struggling to meet multiple objectives when menu planning can use similar tools to design superior menus, balance competing interests and better understand trade-offs.

### Nutrition and consumption

Using optimisation to design school lunch menus might offer meaningful nutritional gains. During the 2014–2015 school year, the average HEI for elementary school lunch menus was 82^(9)^. By comparison, the HEI of lunches brought from home and of the overall diets of children and adolescents was significantly lower, 65 and 54, respectively^(9,33)^. Baseline results for this study were similar to the national average and suggest that the school district is already providing meals which have superior nutritional quality to foods from home or eaten outside of school. However, this research shows that altering the frequency of menu items currently offered could further improve the nutritional quality of lunches. In adults, increases in dietary HEI are associated with improved health outcomes, including a reduced risk of all-cause mortality, and scores above 81 are associated with a reduced risk of obesity^(35,36)^. Optimisation increased the HEI of weekly menus from 84 at baseline to 100, and the HEI of the consumed portion from 77 at baseline to 94. Compared with the Dietary Reference Intake for males 9–13 years old, all optimised menus offered more than the daily requirements for Fe, fibre and Ca^(34)^. As such, instituting optimised menus could provide potential health benefits to students. Optimised menus had greater HEI than baseline menus because they included more fish, legumes and vegetables and less beef, cheese, Na and saturated fat.

While optimisation successfully developed menus with greater nutritional quality, greater nutrients offered did not always result in greater nutrients consumed. Menu planning which incorporates consumption could offer greater nutritional value to students than menus designed to solely enhance nutritional quality. This was clearly illustrated with fibre; the model which maximised nutritional quality (MAX-HEI) offered the most fibre, but potential fibre consumption was greatest for the multi-objective menus with consumption weighting (MIN-DEV10, MIN-DEV30). While this finding might seem to suggest that greater nutritional quality was associated with greater waste, this was not necessarily the case. Our research shows that menus which maximised consumption had higher nutritional quality than baseline menus. This is confirmed by previous research which finds that improved nutritional quality can increase consumption rates for certain meal categories including entrées and vegetables^(37,38)^.

Given the current levels of plate waste in the US NSLP, up to 30 % for certain food categories and the implications of waste, it is imperative to increase school lunch consumption rates. Here, we found that the types of menu items served impact potential consumption rates and that these consumption rates can be improved simultaneously with nutritional quality. Like previous research, we identified considerable variability in consumption rates across meal components and entrée protein type, with vegetables and legumes having some of the lowest consumption rates^(15,37)^. Although similar data collection methods were employed, the consumption rates used in this research were on average nearly 19 % lower than national average estimates^(15)^. This is partially explained by our conservative imputation methods. While differences in consumption rates were stark, the consumption data used for this research were collected from a larger sample than national estimates, offered consistently more conservative estimates and reflected the school district of interest.

### Cost and environmental impacts

After the implementation of Healthy Hunger Free Kids Act, school food authorities reported that the most significant challenge when planning meals was the cost of foods^(39)^. Here, we showed that nutritious lunches with potentially higher student consumption can result in costs within or below budget constraints. This is particularly relevant for the studied school district and the many school districts which run small deficits, as these methods could be used to help maintain budgets while managing other goals. Like previous research, we found that more nutritious lunches did not cost more to produce^(13)^. However, the least cost menu had the lowest HEI and was below the HEI of baseline weekly menus, which suggests a trade-off between minimising cost and maximising nutritional quality.

Previous research using optimisation to design school lunch menus has focused on environmental impacts^(21–23)^. This study additionally integrated concerns pertinent to the US NSLP and was still able to reduce environmental impacts of weekly lunch menus. The reduction observed here most likely resulted from limiting beef and cheese entrées.

The multi-objective models had the lowest environmental impacts. These models also had the greatest nutritional quality apart from the model which maximised nutritional quality. This aligns with previous research which found that improved diet quality is correlated with reduced environmental impacts^(40–42)^. Although this relationship has been previously reported, it is not causal and school districts should be cautious with assumption about this connection because it is not always true for all impacts and foods (i.e. refined sugar has low GWP but is discouraged when assessing diet quality).

### Recommendations

Numerous studies have used optimisation to balance environmental, health, cost and cultural components of diet^(43,44)^. Like this work, these studies found that optimised diets were more plant-based, reduced beef and lamb, in some cases increased fish, and reduced sweets and refined grain bread.

The multi-objective models were most successful in minimising trade-offs and can be used to inform future menu planning. Additionally, the distribution of all model entrée protein types (Fig. 1) provides an outline for recommendations. If our results were extended across the month, entrées containing legumes would appear about once a week, fish entrées would appear every other week, beef and cheese entrées would be offered up to three times a month each and chicken would be offered six times a month.

This outline would require reductions in beef and cheese entrées compared with baseline offerings. Baseline menus offered beef and cheese because they were inexpensive and highly consumed. Even with their high consumption rates, we show that removing or reducing beef and cheese from menus can increase consumption rates by up to about 400 calories per week. Nonetheless, complete removal of beef from menus could be difficult and school districts should consider reducing beef entrées slowly, with the goal of offering it every other week or in smaller portions.

Some of the lowest consumption rates for entrées were for non-pizza vegetarian dishes, yet optimisation models repeatedly selected these options. As such, careful planning of vegetarian entrées, slow or gradual introduction and offering taste tests, interactive cooking demonstrations or other interventions might be necessary to improve consumption rates and acceptance^(45)^. The federal government could support this change by requiring weekly non-pizza vegetarian entrées and pairing changes with grants to support interventions and education.

### Limitations and future directions

Limitations to this research arose from both the tools used to design menus and data availability. Linear programming did not allow for the maximisation of HEI. Using goal programming methods seemed to successfully produce a maximum value for HEI; however, the MIN-DEV10 menu had a higher HEI than the MAX-HEI model. Apart from the goal programming method, a single objective with a wider range of constraints could have been applied. This method, while successful in other research, led to issues including the selection of constraints associated with the objectives and the model finding a solution. In terms of data availability, multiple options for each meal component are available to students daily and the school district does not keep records on student selections. Baseline measurements were based on what was offered to students on the menu, even though certain items might be selected less frequently. Students are more likely to select items that they like which might lead to greater consumption. As such, our baseline average consumption estimate was most likely lower than actual average weekly consumption rates. Similarly, the average nutritional quality of weekly baseline menus was most likely slightly inflated. Without selection data, it is not feasible to assess these values. Ultimately, this work informs menu planning and future research can be designed to incorporate student selection.

Additionally, this research did not include information on produce seasonality or environmental impacts beyond the farm gate. The models selected produce, such as watermelon, which might only be available seasonally. To make the changes suggested, the district will need to consider what products are available in greater quantities and in which seasons. Future analyses could incorporate information on procurement. As mentioned in the methods, the environmental data only included water scarcity and GWP, and the system boundaries for these data did not include waste management. While the impacts of waste management are expected to be lower than those used to produce food, including waste management would provide a more complete picture of the impacts of meals and food waste^(46)^. Also, this analysis did not include impacts relevant to fisheries management such as biodiversity. Including this information is important to better understand the environmental impacts of menu items which contain tuna.

Moving forward, research should assess the impact of implementing optimised menus as has been performed in Swedish schools^(24,25)^. This research could be coupled with various forms of interventions to increase consumption and acceptance of new menus. Research informing implementation will be essential to ensuring that the benefits of optimised menus are realised and that unintended consequences are avoided and could include information to support smaller school districts with fewer resources. Additionally, future modelling studies could consider the shadow prices of constraints, and how constraints could be altered to further improve menus.

## Conclusion

Optimisation and linear programming are effective tools for designing school lunch menus with multiple objectives. These methods allowed for the minimisation of trade-offs across goals and weighting based on priorities for the school district. Compared with weekly baseline menus offered, menus designed with multi-objective models increased potential energy consumption by up to 27 % and HEI by up to 19 %, and reduced costs and environmental impacts by up to 13 % and 71 %, respectively. Improvements were made by reducing the frequency of beef and cheese entrées and increasing the frequency of fish and legume entrées on weekly menus. This work can be extrapolated to monthly menus to provide further direction for school districts and the methods can be employed with different recipes and constraints. In the future, implementation studies should test student acceptance of optimised menus and consider how to increase acceptance of nutritious, low-cost and low-impact items such as legumes.

## Supplementary material

For supplementary material accompanying this paper visit https://doi.org/10.1017/S1368980023000927. click here to view supplementary material
