# Supplementary material for: Improving school lunch menus with multi-objective optimisation: nutrition, cost, consumption and environmental impacts
Source: Public Health Nutr. 2023 May 11;26(8):1715–27. doi: 10.1017/S1368980023000927 (PMC10410403; doi:10.1017/S1368980023000927)
Supplement: Supplementary file 1 [file S1368980023000927sup001.docx]

Improving school lunch menus with multi-objective optimization: nutrition, cost, consumption, and environmental impacts.

Supplemental Information

Contents

[Methods 2](#_Toc126140767)

[Data 2](#_Toc126140768)

[Subcategories 2](#_Toc126140769)

[Global Warming Potential and Water Scarcity 2](#_Toc126140770)

[Linear Programming 2](#_Toc126140771)

[Constraints 2](#_Toc126140772)

[Objective Functions 3](#_Toc126140773)

[SI Table 1. Cost, nutrient content, and frequency of menu items offered during the 2018-2019 school year.^1^ 7](#_Toc126140774)

[SI Table 2. Student Plate Waste Data Imputations. 12](#_Toc126140775)

[SI Table 3. Weekly menus selected by optimization models. 18](#_Toc126140776)

[SI Table 3 Cont. Weekly menus selected by optimization models. 19](#_Toc126140777)

[SI Figure 1. Fruit frequency for baseline and optimized weekly menus. 21](#_Toc126140778)

[SI Figure 2. Vegetable frequency for baseline and optimized weekly menus. 22](#_Toc126140779)

[SI Table 4. Baseline and optimized weekly menus nutrient content, as offered and consumed. 23](#_Toc126140780)

[SI Table 4 Cont. Baseline and optimized weekly menus nutrient content, as offered and consumed. 24](#_Toc126140781)

# Methods

## Data

### Subcategories

Subcategory 2, Flavor Profiles, was developed after running preliminary models and finding that similar foods or flavor profiles such as chicken taco and beef taco were often selected within the same weekly menu. While these entrées contained different protein types, adding Subcategory 2, allowed for a constraint on the repetition of foods with similar ingredients or seasonings. Development of the categories in Subcategory 2 were based on the ingredients and recipes for entrées. This decision was made after conferring with nutrition experts from around the world.

### Consumption

The average grams consumed of each menu item were collected from 7,000 lunches served across 50 days in twelve schools during the 2018-2019 school year from baseline menus offered. When an item was served infrequently and consumption data was limited (< 20 observations), we imputed estimates by combining data on similar items or using the 20^th^ percentile of the proportion consumed from the respective menu item category or entrée subcategory. We used the 20^th^ percentile because we expected consumption rates for these foods to be lower than average. These items were unique (i.e. data on similar options were not available) and served infrequently (≤ ten times per year); therefore, students would be less familiar with these items and more likely to consume less. We consider this to be a highly conservative estimate. Of the 142 menu items included in the analyses, we used the 20^th^ percentile for 21 menu items and combined data for 47 menu items (SI Table 2).

### Global Warming Potential and Water Scarcity

Data were available from Stern et. al (2022) and Heller et. al (2021). Stern et. al (2022) used the ecoinvent 3.6 database to estimate GWP of menu items and Heller et. al (2021) calculated water scarcity using blue water consumption from agricultural irrigation connected to geographic data and the AWARE100 method ^(29)^. The system boundary for GWP was cradle to farm gate and water scarcity only included irrigation. As such, the GWP and water scarcity associated with the end-of-life stages for managing waste (i.e. landfilling) were not included. However, the GWP and water scarcity of producing food that would be wasted were included.

## Linear Programming

Linear programming and optimization are tools to find the best solution among many feasible solutions. The standard form for linear programming is provided here:

$$minimize c_{1}x_{1}+c_{2}x_{2}+\ldots{+ c}_{n}x_{n}$$

$$subject to constraints$$

$$a_{11}x_{1}+a_{12}x_{2}+\ldots+a_{1n}x_{n}\geq b_{1}$$

$$a_{21}x_{1}+a_{22}x_{2}+\ldots+a_{2n}x_{n}\geq b_{2}$$

$\vdots$

$$a_{m1}x_{1}+a_{m2}x_{2}+\ldots+a_{mn}x_{n}\geq b_{m}$$

$$and nonnegativity constraints$$

$$x_{1}\geq0,x_{2}\geq0,\ldots x_{n}\geq0$$

### Constraints

Variety in school meals is important for nutrition and student acceptance, therefore we included constraints on the number of times a food could be served within the week. Each optimized weekly menu included five servings of each meal component (entrée, fruit, and milk), except for vegetables which could have up to ten servings per week, or two vegetables per lunch. This follows general practice of the school district which provides up to two vegetables options each day to meet NSLP requirements. No entrée was repeated more than once throughout the week and no more than two foods from Subcategory 1, Protein Types or one food from Subcategory 2, Flavor Profiles could be selected. This limited the model from repeatedly choosing similar foods. An exception for fish (Subcategory 1) was made; only one fish item could be selected each week because fish is not well liked by students ^(32)^. Each fruit and vegetable could be repeated up to two times per week and there was no constraint on repetitions of milk type.

### Objective Functions

Prior to developing the objective functions used in this research, preliminary objective functions were developed to understand which variables to maximize or minimize. Analyses considered maximizing grams consumed and specific nutrients relevant to childhood populations.

The modeled menus with objective functions which maximized grams consumed selected the largest foods by weight. This objective function maximized grams consumed which was not relative to the starting weights of menu items and therefore did not produce the desired goals. For example, a serving of Mandarin orange chicken with brown rice weighed about 241 grams and a hot dog on a bun with popcorn weighed about 100 grams. Students could eat 90% of the hot dog entrée about 90 g but if they ate 90 g of the Mandarin chicken entrée this would only account for about 37% of the entrée. Due to this limitation, the proportion consumed was applied to calories and dollar value of the meal and these values were maximized.

Other preliminary objective functions maximized calcium, iron, and fiber. These objective functions produced menus which performed poorly for other nutrients and therefore a combined objective function which is described below was used to maximize nutritional quality of menus.

The following section describes the objective functions used for this research.

The first objective functions (O1-O2) developed weekly menus with the greatest potential consumption. These functions maximize the total energy (kcal) consumed and total value (USD) consumed of a weekly menu and are expressed mathematically by:

Equation O1.

$$MAX f(CALCON)=\sum_{i=1}^{142} {x_{i}*CAL}_{i}*{CON}_{i}$$

Equation O2.

$$MAX f(COSTCON)=\sum_{i=1}^{142} {x_{i}*COST}_{i}*{CON}_{i}$$

where, *i*= 1, …, 142 represents the menu items, *x_i_* is the decision variable and represents the number of servings of each menu item *i* selected, and *CAL_i_*, *CON_i_*, and *COST_i_* represent the energy (kcal), consumption rates, and value (USD) per serving of each menu item *i*, respectively. For example, per serving, a banana contains 105 kcal, costs 0.12 USD, and on average, the consumption rate is 60%. Therefore, the *CALCON* and *COSTCON* for a menu that only contained one banana would be 63 kcal and 0.07 USD, respectively. The objective functions find which combination of menu items yield the maximum total *CALCON* and *COSTCON*. We were interested in maximizing the value consumed to understand how efficient the district was with its budget.

The next objective function (O3) finds the least cost weekly menu. This function minimized the total cost of a weekly menu served and is expressed mathematically by:

Equation O3.

$$MIN f\left( COST \right)=\sum_{i=1}^{142} {x_{i}*COST}_{i}$$

The next objective function (O4) maximized the nutritional quality of a weekly menu. While we measured the nutritional quality of menus with the HEI, the equation for HEI is nonlinear, which means that it cannot be used as an objective function in linear programming. While there are nonlinear methods for optimization, they are computationally intensive and do not guarantee that the global minimum or maximum is found. Instead, to use a linear function, we used the components and weighting of HEI with a method of goal programming which allows for the consideration of multiple variables simultaneously.

In goal programming, the objective is to minimize the sum of the relative deviations from predetermined goals. There are 13 components in the HEI, therefore, we had 13 separate goals in the nutrition objective function (O4). The goals were to maximize the components to encourage (total fruit, whole fruit, total vegetable, greens and beans, whole grains, dairy, total protein foods, seafood and plant proteins, and fatty acids) and to minimize the components to discourage (refined grains, sodium, added sugar, and saturated fats). For each component, we found the maximum or minimum possible value for a weekly lunch menu subject to constraints. For example, the goal for the greens and beans component was five cup eq., which was the maximum possible amount of dark green vegetables and legumes in a weekly menu. The objective function minimized the deviations from these goals and all deviations were standardized to manage varying scales and units by dividing by the initial goal. We then weighted each relative deviation by the maximum possible score per component in the HEI (Table 1). This function is expressed mathematically by:

Equation O4.

$$MIN f\left( dHEI \right)=\sum_{i=1}^{142} \left[ \sum_{c=1}^{9} \left( \frac{{{{max}_{c}-(x}_{i}*encourage}_{ic})}{{max}_{c}}*W_{c} \right)+\sum_{c=1}^{4} \left( \frac{{{(x}_{i}*discourage}_{ic})-{min}_{c}}{{min}_{c}}*W_{c} \right) \right]$$

where, *c*= 1, …, 9 represents the components to encourage, *c*= 1, …, 4 represents the components to discourage, *max_c_* is the maximum value per weekly menu of each component to encourage, *encourage_ic_* is the amount of each component in cup eq., oz. eq., or grams for each menu item, *W_c_* is the weight of each component based on the maximum score in HEI, *min_c_* is the minimum value per weekly menu of each component to discourage, and *discourage_ic_* is the amount of each component in cup eq., oz. eq., or grams for each food item.

We recognize that this method of maximizing nutritional quality does not align entirely with the HEI, as it encourages menus to go beyond the cutoff values for each component. Although we were unable to use the HEI as an objective function, we still assessed the HEI of each resulting menu and provide this information in the results.

Goal programming was also used in our final objective functions (O5-O7) to combine consumption, cost, nutritional quality, and environmental goals. We refer to the following objective functions as the *multi-objective functions* because they consider multiple disparate goals. As described above, these objective functions minimized the sum of the relative deviations from each goal. The goals for consumption, cost, and nutritional quality were calculated using the objective functions O1, O3, and O4, respectively. The goals for the environmental outcomes were the minimum global warming potential and water scarcity per weekly menu as determined in previous research ^(33)^. For each goal, we applied weights to express the potential importance to a school district. This function is expressed mathematically by:

Equation O5.

$$MIN f\left( DEV \right)=\sum_{i=1}^{142} \left[ \left( \frac{{{{CONCAL}_{max}-(x}_{i}*CAL}_{i}*{CON}_{i})}{{CONCAL}_{max}}*W_{cal} \right)+\sum_{g=1}^{4} \left( \frac{{{(x}_{i}*z}_{ig})-{min}_{g}}{{min}_{g}}*W_{g} \right) \right]$$

where, *CONCAL_max_* is the maximum calories consumed in a weekly menu, *W_cal_* is the weight applied to the relative deviation of the calories consumed goal, *g*= 1, …, 4 are the four goals which are minimized (cost, nutritional quality, global warming potential, and water scarcity), z*_ig_* is the coefficient for each food item for each goal, *min_g_* is the minimum value per weekly menu of each goal, and *W_g_* is the weight applied to each of the relative deviations per goal. In this equation, *CONCAL* is summed separately from the other goals because it is subtracting from the maximum whereas the other goals subtract the minimum.

In objective function O5, we applied equal weights to each goal (*W_cal_* = 1 and *W_1-4_* = 1) and in objective function O6 we applied a weight of ten to the calories consumed goal. In objective function O7 we applied a weight of 30 to the calories consumed goal, because increasing consumption is the primary goal of the district and reducing environmental impacts has been associated with improved nutritional quality ^(34)^.

# SI Table 1. Cost, nutrient content, and frequency of menu items offered during the 2018-2019 school year.^1^

| Menu Item Category | Entrée  Subcategory  1 | Entrée  Subcategory  2 | Menu Item Name^2^ | Cost (USD) | Energy (kcal) | Sodium (mg) | Sat. Fat (g) | Frequency Offered^3^ (%) |
| --- | --- | --- | --- | --- | --- | --- | --- | --- |
| Entrée | Beef | Tomato Salsa | Beef Burrito | 0.74 | 455 | 610 | 9.50 | 1.4 |
| Entrée | Beef | Tomato Salsa | Beef Nachos | 0.52 | 375 | 595 | 10.50 | 1.4 |
| Entrée | Beef | Tomato Salsa | Beef Taco | 0.56 | 435 | 480 | 11.00 | 2.0 |
| Entrée | Beef |  | Cheeseburger | 0.34 | 301 | 594 | 4.50 | 4.3 |
| Entrée | Beef |  | Hamburger | 0.29 | 266 | 489 | 3.50 | 6.9 |
| Entrée | Beef |  | Hot Dog on a Bun with Popcorn | 0.99 | 370 | 555 | 6.43 | 0.5 |
| Entrée | Beef | Cheese | Meatball Pizza | 0.74 | 305 | 782 | 5.00 | 1.3 |
| Entrée | Beef |  | Meatball Sandwich | 0.71 | 376 | 724 | 7.50 | 2.8 |
| Entrée | Beef |  | Meatballs and Garlic Bread | 0.37 | 240 | 410 | 4.50 | 0.4 |
| Entrée | Beef |  | Pepper Jack Cheeseburger | 0.71 | 376 | 679 | 9.50 | 1.3 |
| Entrée | Beef | Pasta | Spaghetti with Marinara and Meatballs | 0.91 | 380 | 970 | 7.50 | 3.9 |
| Entrée | Beef |  | Steak and Cheese Croissant | 0.46 | 390 | 810 | 9.00 | 1.4 |
| Entrée | Beef |  | Steak and Cheese Sandwich | 1.15 | 373 | 692 | 5.47 | 1.6 |
| Entrée | Chicken | Barbecue | BBQ Chicken Drumstick with Brown Rice | 0.92 | 300 | 675 | 2.15 | 0.4 |
| Entrée | Chicken | Barbecue | BBQ Chicken Drumstick with Cornbread | 1.28 | 370 | 765 | 2.65 | 0.9 |
| Entrée | Chicken | Barbecue | BBQ Chicken Drumstick with Pesto Pasta | 2.83 | 695 | 1305 | 11.65 | 0.6 |
| Entrée | Chicken | Cheese | BBQ Chicken Pizza | 0.74 | 300 | 786 | 4.75 | 2.4 |
| Entrée | Chicken | Salad | Buffalo Chicken Garden Salad | 0.82 | 231 | 485 | 3.75 | 1.5 |
| Entrée | Chicken | Cheese | Buffalo Chicken Pizza | 0.74 | 290 | 903 | 4.75 | 2.5 |
| Entrée | Chicken | Soy/Orange | Chicken Banh Mi | 0.65 | 323 | 896 | 2.44 | 0.9 |
| Entrée | Chicken | Tomato Salsa | Chicken Burrito | 0.86 | 465 | 750 | 8.00 | 0.5 |
| Entrée | Chicken | Salad | Chicken Caesar Salad | 0.87 | 400 | 688 | 7.00 | 2.1 |
| Entrée | Chicken | Tomato Salsa | Chicken Nachos | 0.64 | 385 | 735 | 9.00 | 1.9 |
| Entrée | Chicken |  | Chicken Parmesan Sandwich | 0.88 | 286 | 644 | 1.50 | 0.3 |
| Entrée | Chicken | Cheese | Chicken Quesadilla | 1.29 | 380 | 857 | 5.79 | 0.9 |
| Entrée | Chicken | Tomato Salsa | Chicken Taco | 0.68 | 445 | 620 | 9.50 | 0.6 |
| Entrée | Chicken |  | Chicken Tenders with Cheese Bites | 1.20 | 281 | 537 | 2.40 | 0.8 |
| Entrée | Chicken |  | Chicken Tenders with Dinner Roll | 0.55 | 284 | 529 | 1.40 | 0.3 |
| Entrée | Chicken |  | Crispy Chicken Sandwich | 0.71 | 344 | 679 | 2.00 | 2.6 |
| Entrée | Chicken | All Spice/Turmeric | Curry Chicken with Biscuit | 0.68 | 198 | 656 | 1.95 | 0.0 |
| Entrée | Chicken | All Spice/Turmeric | Curry Chicken with Brown Rice | 0.57 | 185 | 385 | 1.00 | 1.0 |
| Entrée | Chicken | All Spice/Turmeric | Curry Chicken with Flatbread | 0.70 | 285 | 715 | 2.00 | 0.0 |
| Entrée | Chicken |  | Grilled Chicken Sandwich | 0.82 | 284 | 579 | 0.50 | 0.6 |
| Entrée | Chicken | All Spice/Turmeric | Jerk Chicken Drumstick with Brown Rice and Beans | 0.89 | 159 | 401 | 2.00 | 0.8 |
| Entrée | Chicken | Soy/Orange | Mandarin Orange Chicken with Brown Rice | 0.77 | 230 | 280 | 0.50 | 1.5 |
| Entrée | Chicken | Barbecue | Pulled BBQ Chicken Sandwich | 0.55 | 339 | 862 | 2.25 | 0.6 |
| Entrée | Chicken | Barbecue | Pulled BBQ Chicken with Cornbread | 0.80 | 308 | 593 | 1.75 | 0.9 |
| Entrée | Chicken | All Spice/Turmeric | Pulled Jerk Chicken with Biscuit | 0.60 | 190 | 500 | 2.05 | 0.1 |
| Entrée | Chicken | All Spice/Turmeric | Pulled Jerk Chicken with Cornbread | 0.85 | 247 | 319 | 1.60 | 0.0 |
| Entrée | Chicken | All Spice/Turmeric | Seasoned Chicken and Brown Rice | 0.40 | 210 | 330 | 2.00 | 0.4 |
| Entrée | Chicken | Soy/Orange | Teriyaki Chicken with Lo Mein Noodles | 0.64 | 245 | 590 | 3.00 | 1.5 |
| Entrée | Chicken | Soy/Orange | Teriyaki Chicken with Vegetable Fried Rice | 0.76 | 399 | 504 | 1.00 | 1.8 |
| Entrée | Fish |  | Baked Fish and Cornbread | 1.32 | 410 | 560 | 2.50 | 0.0 |
| Entrée | Fish |  | Baked Fish in Chip with Brown Rice | 0.66 | 310 | 260 | 1.00 | 1.5 |
| Entrée | Fish |  | Baked Fish Sandwich | 1.07 | 441 | 829 | 3.00 | 1.6 |
| Entrée | Fish | Tomato Salsa | Fish Burrito | 1.12 | 525 | 680 | 7.00 | 0.1 |
| Entrée | Fish | Tomato Salsa | Fish Taco | 0.94 | 505 | 550 | 8.50 | 0.6 |
| Entrée | Fish | Salad | Garden Salad and Tuna | 0.55 | 147 | 280 | 0.98 | 0.6 |
| Entrée | Fish |  | Tuna with Pita Bread | 0.46 | 244 | 460 | 0.98 | 0.1 |
| Entrée | Turkey | Salad | Chef Salad with Turkey and Cheese and Pita | 1.04 | 346 | 850 | 3.25 | 2.0 |
| Entrée | Turkey |  | Roast Turkey with Cornbread and Gravy | 1.12 | 277 | 812 | 1.34 | 0.6 |
| Entrée | Turkey |  | Turkey and Cheese Sandwich | 1.14 | 267 | 841 | 1.50 | 0.6 |
| Entrée | Vegetarian | Tomato Salsa | Bean Burrito | 0.66 | 459 | 1048 | 6.10 | 3.3 |
| Entrée | Vegetarian | Tomato Salsa | Bean Taco | 0.48 | 439 | 918 | 7.60 | 1.4 |
| Entrée | Vegetarian | Cheese | Broccoli and Cheddar Croissant | 0.96 | 420 | 870 | 11.00 | 1.6 |
| Entrée | Vegetarian | Cheese | Cheese Bites | 0.74 | 70 | 135 | 1.00 | 2.3 |
| Entrée | Vegetarian | Cheese | Cheese Pizza | 0.74 | 270 | 710 | 4.50 | 9.9 |
| Entrée | Vegetarian | Cheese | Cheese Quesadilla | 0.97 | 389 | 724 | 10.31 | 0.8 |
| Entrée | Vegetarian | Cheese | Grilled Cheese | 0.37 | 282 | 696 | 4.00 | 2.5 |
| Entrée | Vegetarian |  | Hummus, Vegetables, Cheese, and Flatbread | 1.27 | 497 | 948 | 7.50 | 0.4 |
| Entrée | Vegetarian | Cheese | Macaroni and Cheese | 0.46 | 213 | 412 | 4.80 | 1.1 |
| Entrée | Vegetarian | Tofu | Marinated Tofu Banh Mi | 0.39 | 206 | 444 | 0.28 | 0.9 |
| Entrée | Vegetarian | Tofu | Marinated Tofu with Brown Rice | 0.28 | 140 | 190 | 0.28 | 0.3 |
| Entrée | Vegetarian | Tofu | Marinated Tofu with Cornbread | 0.64 | 210 | 280 | 0.78 | 0.0 |
| Entrée | Vegetarian | Tofu | Marinated Tofu with Lo Mein Noodles | 0.29 | 190 | 450 | 2.28 | 1.3 |
| Entrée | Vegetarian | Tofu | Marinated Tofu with Vegetable Fried Rice | 0.41 | 344 | 364 | 0.28 | 0.6 |
| Entrée | Vegetarian | Pasta | Pasta with Pesto | 1.99 | 475 | 630 | 9.50 | 0.0 |
| Entrée | Vegetarian |  | Peanut Butter and Jelly Sandwich | 0.57 | 300 | 280 | 3.00 | 0.5 |
| Entrée | Vegetarian | Pasta | Spaghetti with Marinara and Cheese | 0.72 | 240 | 680 | 3.50 | 1.5 |
| Entrée | Vegetarian | Cheese | Spinach Quesadilla | 1.34 | 414 | 804 | 8.66 | 0.5 |
| Entrée | Vegetarian |  | Three Bean Chili with Cornbread | 0.72 | 391 | 546 | 7.10 | 0.3 |
| Entrée | Vegetarian |  | Three Bean Chili with Tortilla Chips | 0.38 | 381 | 571 | 7.60 | 0.6 |
| Entrée | Vegetarian | Cheese | Toasted Cheese Sandwich and Three Bean Chili | 0.45 | 378 | 857 | 3.60 | 1.1 |
| Entrée | Vegetarian | All Spice/Turmeric | Vegetable Curry with Brown Rice | 0.47 | 333 | 642 | 4.76 | 0.4 |
| Entrée | Vegetarian | Cheese | Veggie Pizza | 0.74 | 267 | 600 | 4.39 | 0.4 |
| Entrée | Vegetarian | Cheese | White Garlic Pizza | 0.74 | 300 | 650 | 6.00 | 3.5 |
| Entrée | Vegetarian |  | Yogurt Grab and Go | 0.81 | 420 | 310 | 1.00 | 0.3 |
| Fruit |  |  | Apple, Fresh | 0.23 | 72 | 1 | 0.00 | 38.0 |
| Fruit |  |  | Applesauce | 0.23 | 51 | 2 | 0.00 | 0.1 |
| Fruit |  |  | Assorted Chilled Fruit Cups | 0.23 | 61 | 4 | 0.00 | 0.1 |
| Fruit |  |  | Banana, Fresh | 0.12 | 105 | 1 | 0.00 | 18.0 |
| Fruit |  |  | Blueberries, Frozen | 0.25 | 40 | 2 | 0.00 | 0.1 |
| Fruit |  |  | Craisins | 0.25 | 110 | 0 | 0.00 | 0.1 |
| Fruit |  |  | Dried Fruit | 0.31 | 120 | 25 | 0.00 | 0.0 |
| Fruit |  |  | Mandarin Oranges, Canned | 0.22 | 60 | 10 | 0.00 | 3.5 |
| Fruit |  |  | Orange, Medium | 0.23 | 62 | 0 | 0.00 | 21.0 |
| Fruit |  |  | Peaches, Canned | 0.26 | 60 | 5 | 0.00 | 1.0 |
| Fruit |  |  | Peaches, Fresh | 0.70 | 51 | 0 | 0.00 | 2.2 |
| Fruit |  |  | Pear, Fresh | 0.25 | 101 | 1 | 0.00 | 9.0 |
| Fruit |  |  | Raisins | 0.18 | 129 | 4 | 0.00 | 0.1 |
| Fruit |  |  | Strawberries, Frozen | 0.72 | 25 | 0 | 0.00 | 0.6 |
| Fruit |  |  | Strawberry Cups | 0.72 | 90 | 0 | 0.00 | 0.6 |
| Fruit |  |  | Strawberry Pouch | 0.33 | 100 | 70 | 1.00 | 0.0 |
| Fruit |  |  | Tropical Fruit Blend, Canned | 0.27 | 80 | 0 | 0.00 | 1.0 |
| Fruit |  |  | Watermelon, Fresh | 0.07 | 23 | 1 | 0.00 | 4.6 |
| Milk |  |  | 1% Plain Milk | 0.21 | 110 | 130 | 1.50 | 50.0 |
| Milk |  |  | Lactaid Milk, Fat Free | 0.46 | 90 | 125 | 0.00 | 5.8 |
| Milk |  |  | Skim Chocolate Milk | 0.22 | 120 | 180 | 0.00 | 43.0 |
| Milk |  |  | Skim Plain Milk | 0.21 | 90 | 130 | 0.00 | 7.0 |
| Vegetable |  |  | Baked Potato | 0.12 | 139 | 307 | 1.14 | 0.5 |
| Vegetable |  |  | Beans, Baked Beans | 0.13 | 119 | 140 | 0.90 | 0.5 |
| Vegetable |  |  | Beans, Chickpea Salad | 0.13 | 151 | 500 | 0.68 | 3.0 |
| Vegetable |  |  | Beans, Sizzlin' Black Beans | 0.12 | 124 | 628 | 0.10 | 2.9 |
| Vegetable |  |  | Broccoli, Raw | 0.20 | 15 | 14 | 0.00 | 1.9 |
| Vegetable |  |  | Broccoli, Roasted | 0.20 | 43 | 33 | 0.00 | 5.3 |
| Vegetable |  |  | Broccoli, Steamed | 0.20 | 26 | 22 | 0.00 | 2.0 |
| Vegetable |  |  | Butternut Squash, Roasted | 0.23 | 92 | 7 | 0.00 | 0.0 |
| Vegetable |  |  | Caesar Salad | 0.55 | 270 | 358 | 5.00 | 0.0 |
| Vegetable |  |  | Carrots, Baby, Ranch | 0.23 | 65 | 115 | 0.00 | 6.8 |
| Vegetable |  |  | Carrots, Roasted | 0.08 | 31 | 159 | 0.33 | 9.8 |
| Vegetable |  |  | Cauliflower, Raw | 0.10 | 51 | 80 | 0.00 | 0.0 |
| Vegetable |  |  | Cauliflower, Roasted | 0.10 | 27 | 188 | 0.33 | 1.8 |
| Vegetable |  |  | Celery Sticks, Ranch | 0.20 | 42 | 113 | 0.00 | 1.6 |
| Vegetable |  |  | Cherry Tomatoes, Ranch | 0.09 | 48 | 60 | 0.00 | 1.6 |
| Vegetable |  |  | Coleslaw | 0.14 | 47 | 71 | 0.44 | 2.0 |
| Vegetable |  |  | Corn on the Cob, 3" | 0.20 | 90 | 0 | 0.00 | 2.9 |
| Vegetable |  |  | Corn, Tomato Salsa Street | 0.18 | 161 | 166 | 1.40 | 0.0 |
| Vegetable |  |  | Corn, Sweet Kernels | 0.08 | 96 | 0 | 0.00 | 11.6 |
| Vegetable |  |  | Cucumber Slices, Ranch | 0.12 | 37 | 55 | 0.00 | 4.5 |
| Vegetable |  |  | Diced Potato Hash Browns | 0.46 | 218 | 221 | 3.78 | 0.5 |
| Vegetable |  |  | Green Beans, Steamed | 0.08 | 35 | 0 | 0.00 | 0.0 |
| Vegetable |  |  | Marinara Cup | 0.28 | 40 | 200 | 0.00 | 0.0 |
| Vegetable |  |  | Mashed Potatoes, Recipe 1 | 0.25 | 132 | 394 | 1.00 | 0.8 |
| Vegetable |  |  | Mashed Potatoes, Recipe 2 | 0.31 | 110 | 340 | 1.00 | 0.4 |
| Vegetable |  |  | Onions, Cooked | 0.04 | 29 | 100 | 0.16 | 0.1 |
| Vegetable |  |  | Parsnips | 0.25 | 75 | 201 | 0.35 | 0.0 |
| Vegetable |  |  | Peas | 0.08 | 68 | 265 | 0.04 | 0.4 |
| Vegetable |  |  | Peppers, Cooked | 0.17 | 16 | 86 | 0.15 | 0.1 |
| Vegetable |  |  | Peppers, Ranch | 0.22 | 42 | 57 | 0.03 | 0.5 |
| Vegetable |  |  | Plantain Slices | 0.56 | 300 | 0 | 1.00 | 2.5 |
| Vegetable |  |  | Potato Wedges, Seasoned, Ketchup | 0.47 | 130 | 225 | 0.50 | 3.3 |
| Vegetable |  |  | Red Potatoes, Roasted | 0.10 | 97 | 157 | 0.60 | 0.0 |
| Vegetable |  |  | Romaine, Raw | 0.08 | 5 | 2 | 0.00 | 2.3 |
| Vegetable |  |  | Side Salad, Citrus Spinach | 0.19 | 65 | 73 | 0.00 | 2.9 |
| Vegetable |  |  | Side Salad, Cucumber and Tomato | 0.15 | 52 | 51 | 0.30 | 1.0 |
| Vegetable |  |  | Side Salad, Garden Side | 0.19 | 43 | 60 | 0.00 | 7.6 |
| Vegetable |  |  | Side Salad, Kale and Apple | 0.17 | 244 | 151 | 0.97 | 0.0 |
| Vegetable |  |  | Spinach, Cooked | 0.37 | 21 | 248 | 0.04 | 0.0 |
| Vegetable |  |  | Sweet Potato Wedges | 0.31 | 150 | 125 | 2.00 | 16.0 |
| Vegetable |  |  | Turnips, Roasted | 0.23 | 38 | 76 | 0.00 | 0.0 |
| Vegetable |  |  | Yuca, Baked | 0.47 | 160 | 170 | 1.00 | 0.5 |
| Vegetable |  |  | Yucca Fries, Ketchup | 0.51 | 170 | 85 | 1.00 | 2.8 |

^1^All nutrient content and cost data are per serving.

^2^Menu items names are summaries of the menu item and do not list all ingredients included. Tacos included cheese, rice, and tomato salsa. Quesadillas were served with tomato salsa. All salads included ranch dressing, and entrée salads included a side of pita bread. Spaghetti, pasta, fish sandwich, jerk chicken drumstick, pulled BBQ chicken sandwich, and three bean chili are served with cheese. The yogurt grab and go included fruited yogurt, a mozzarella cheese stick, graham cracker sticks and granola.

^3^Baseline frequencies were assessed over the 2018-2019 school year and are presented as the percent of all items served by menu item category.

# SI Table 2. Student Plate Waste Data Imputations.

| Subcategory  1 | Menu Item | Imputed (Yes = 1, No = 0) | Sample Size Without Imputation | Sample Size with Imputation | Imputation Methods |
| --- | --- | --- | --- | --- | --- |
| Beef | Beef Burrito | 0 | 370 | NA | NA |
| Beef | Beef Nachos | 0 | 33 | NA | NA |
| Beef | Beef Taco | 0 | 90 | NA | NA |
| Beef | Cheeseburger | 0 | 463 | NA | NA |
| Beef | Hamburger | 1 | 7 | 470 | Combined Hamburger and Cheeseburger |
| Beef | Hot Dog on a Bun with Popcorn | 0 | 62 | NA | NA |
| Beef | Meatball Pizza | 1 | 0 | 130 | Used Pepperoni Pizza |
| Beef | Meatball Sandwich | 0 | 157 | NA | NA |
| Beef | Meatballs and Garlic Bread | 0 | 30 | NA | NA |
| Beef | Pepper Jack Cheeseburger | 0 | 463 | NA | NA |
| Beef | Spaghetti with Marinara and Meatballs | 0 | 477 | NA | NA |
| Beef | Steak and Cheese Croissant | 0 | 32 | NA | NA |
| Beef | Steak and Cheese Sandwich | 0 | 31 | NA | NA |
| Chicken | BBQ Chicken Drumstick with Brown Rice | 0 | 108 | NA | NA |
| Chicken | BBQ Chicken Drumstick with Cornbread | 1 | 0 | 108 | Used BBQ Chicken Drumstick with Brown Rice |
| Chicken | BBQ Chicken Drumstick with Pesto Pasta | 1 | 0 | 147 | Used Chicken with Pasta, sauce not specified |
| Chicken | BBQ Chicken Pizza | 0 | 29 | NA | NA |
| Chicken | Buffalo Chicken Garden Salad | 1 | 0 | 40 | Used Chef Salad with Chicken |
| Chicken | Buffalo Chicken Pizza | 1 | 0 | 29 | Used BBQ Chicken Pizza |
| Chicken | Chicken Banh Mi | 1 | 0 | 23 | Used Teriyaki Chicken with Vegetable Fried Rice |
| Chicken | Chicken Burrito | 1 | 0 | 118 | Combined Beef Burrito and Chicken Taco |
| Chicken | Chicken Caesar Salad | 1 | 0 | 40 | Used Chef Salad with Chicken |
| Chicken | Chicken Nachos | 1 | 0 | 33 | Used Beef Nachos |
| Chicken | Chicken Parmesan Sandwich | 1 | 0 | 62 | Used Chicken Melt and Chicken Parmesan with Pasta |
| Chicken | Chicken Quesadilla | 0 | 224 | NA | NA |
| Chicken | Chicken Taco | 1 | 0 | 90 | Used Beef Taco |
| Chicken | Chicken Tenders with Cheese Bites | 1 | 0 | 250 | Used Chicken Tenders with Dinner Roll and Cheese Bites |
| Chicken | Chicken Tenders with Dinner Roll | 0 | 170 | NA | NA |
| Chicken | Crispy Chicken Sandwich | 0 | 206 | NA | NA |
| Chicken | Curry Chicken with Biscuit | 1 | 0 | 80 | Used Curry Chicken with Brown Rice |
| Chicken | Curry Chicken with Brown Rice | 0 | 80 | NA | NA |
| Chicken | Curry Chicken with Flatbread | 1 | 0 | 80 | Used Curry Chicken with Brown Rice |
| Chicken | Grilled Chicken Sandwich | 0 | 35 | NA | NA |
| Chicken | Jerk Chicken Drumstick with Brown Rice and Beans | 1 | 0 | 271 | Combined Seasoned Chicken and Brown Rice and Curry Chicken with Brown Rice |
| Chicken | Mandarin Orange Chicken with Brown Rice | 1 | 11 | 21 | Combined Mandarin Orange Chicken with Brown Rice and General Tso's Chicken |
| Chicken | Pulled BBQ Chicken Sandwich | 0 | 46 | NA | NA |
| Chicken | Pulled BBQ Chicken with Cornbread | 1 | 0 | 46 | Used Pulled BBQ Chicken Sandwich |
| Chicken | Pulled Jerk Chicken with Biscuit | 1 | 0 | 271 | Combined Seasoned Chicken and Brown Rice and Curry Chicken with Brown Rice |
| Chicken | Pulled Jerk Chicken with Cornbread | 1 | 0 | 271 | Combined Seasoned Chicken and Brown Rice and Curry Chicken with Brown Rice |
| Chicken | Seasoned Chicken and Brown Rice | 0 | 191 | NA | NA |
| Chicken | Teriyaki Chicken with Lo Mein Noodles | 0 | 281 | NA | NA |
| Chicken | Teriyaki Chicken with Vegetable Fried Rice | 0 | 23 | NA | NA |
| Fish | Baked Fish and Cornbread | 1 | 0 | 75 | Used Baked Fish Sandwich |
| Fish | Baked Fish in Chip with Brown Rice | 1 | 0 | 75 | Used Baked Fish Sandwich |
| Fish | Baked Fish Sandwich | 0 | 73 | NA | NA |
| Fish | Fish Burrito | 1 | 0 | 75 | Used Baked Fish Sandwich |
| Fish | Fish Taco | 1 | 0 | 75 | Used Baked Fish Sandwich |
| Fish | Garden Salad and Tuna | 1 | 0 | 40 | Used Chef Salad with Chicken |
| Fish | Tuna with Pita Bread | 1 | 0 | 75 | Used Baked Fish Sandwich |
| Fruit | Apple, Fresh | 0 | 2336 | NA | NA |
| Fruit | Applesauce | 1 | 1 | 104 | Combined Sliced Apples and Applesauce |
| Fruit | Assorted Chilled Fruit Cups | 1 | 0 | 20th | 20th percentile fruit |
| Fruit | Banana, Fresh | 0 | 158 | NA | NA |
| Fruit | Blueberries, Frozen | 1 | 0 | 78 | Used Strawberry cups |
| Fruit | Craisins | 1 | 0 | 20th | 20th percentile fruit |
| Fruit | Dried Fruit | 1 | 0 | 20th | 20th percentile fruit |
| Fruit | Mandarin Oranges, Canned | 1 | 0 | 384 | Combined Clementine and Tangerine |
| Fruit | Orange, Medium | 0 | 930 | NA | NA |
| Fruit | Peaches, Canned | 1 | 0 | 20th | 20th percentile fruit |
| Fruit | Peaches, Fresh | 1 | 0 | 283 | Used Pear, Fresh |
| Fruit | Pear, Fresh | 0 | 283 | NA | NA |
| Fruit | Raisins | 1 | 0 | 20th | 20th percentile fruit |
| Fruit | Strawberries, Frozen | 0 | 83 | NA | NA |
| Fruit | Strawberry Cups | 0 | 83 | NA | NA |
| Fruit | Strawberry Pouch | 1 | 0 | 20th | 20th percentile fruit |
| Fruit | Tropical Fruit Blend, Canned | 1 | 0 | 20th | 20th percentile fruit |
| Fruit | Watermelon, Fresh | 0 | 483 | NA | NA |
| Milk | 1% Plain Milk | 0 | 2278 | NA | NA |
| Milk | Lactaid Milk, Fat Free | 1 | 13 | 180 | Combined Lactaid Milk and Skim Milk |
| Milk | Skim Chocolate Milk | 0 | 1271 | NA | NA |
| Milk | Skim Plain Milk | 0 | 167 | NA | NA |
| Turkey | Chef Salad with Turkey and Cheese and Pita | 1 | 0 | 40 | Used Chef Salad with Chicken |
| Turkey | Roast Turkey with Cornbread and Gravy | 1 | 0 | 41 | Used Turkey and Cheese Sandwich |
| Turkey | Turkey and Cheese Sandwich | 0 | 41 | NA | NA |
| Vegetable | Baked Potato | 1 | 0 | 56 | Used Potato Wedges |
| Vegetable | Beans, Baked Beans | 0 | 123 | NA | NA |
| Vegetable | Beans, Chickpea Salad | 0 | 20 | NA | NA |
| Vegetable | Beans, Sizzlin' Black Beans | 0 | 188 | NA | NA |
| Vegetable | Broccoli, Raw | 0 | 36 | NA | NA |
| Vegetable | Broccoli, Roasted | 0 | 139 | NA | NA |
| Vegetable | Broccoli, Steamed | 0 | 645 | NA | NA |
| Vegetable | Butternut Squash, Roasted | 1 | 0 | 20th | 20th percentile vegetable |
| Vegetable | Caesar Salad | 1 | 0 | 465 | Used Salad Unspecified |
| Vegetable | Carrots, Baby, Ranch | 0 | 304 | NA | NA |
| Vegetable | Carrots, Roasted | 0 | 348 | NA | NA |
| Vegetable | Cauliflower, Raw | 1 | 0 | 20th | 20th percentile vegetable |
| Vegetable | Cauliflower, Roasted | 1 | 0 | 139 | Used Broccoli |
| Vegetable | Celery Sticks, Ranch | 0 | 50 | NA | NA |
| Vegetable | Cherry Tomatoes, Ranch | 0 | 230 | NA | NA |
| Vegetable | Coleslaw | 0 | 39 | NA | NA |
| Vegetable | Corn on the Cob, 3" | 0 | 254 | NA | NA |
| Vegetable | Corn, Mexican Street | 0 | 64 | NA | NA |
| Vegetable | Corn, Sweet Kernels | 0 | 510 | NA | NA |
| Vegetable | Cucumber Slices, Ranch | 0 | 1130 | NA | NA |
| Vegetable | Diced Potato Hash Browns | 1 | 0 | 56 | Combined Potato Wedges and Roasted Potatoes |
| Vegetable | Green Beans, Steamed | 0 | 66 | NA | NA |
| Vegetable | Marinara Cup | 1 | 0 | 20th | 20th percentile vegetable |
| Vegetable | Mashed Potatoes, Recipe 1 | 1 | 0 | 576 | Combined Potato Wedges and Roasted Potatoes and Sweet Potato Fries |
| Vegetable | Mashed Potatoes, Recipe 2 | 1 | 0 | 576 | Combined Potato Wedges and Roasted Potatoes and Sweet Potato Fries |
| Vegetable | Onions, Cooked | 1 | 0 | 20th | 20th percentile vegetable |
| Vegetable | Parsnips | 1 | 0 | 20th | 20th percentile vegetable |
| Vegetable | Peas | 0 | 290 | NA | NA |
| Vegetable | Peppers, Cooked | 1 | 0 | 183 | Used Peppers, Unspecified if cooked |
| Vegetable | Peppers, Ranch | 0 | 183 | NA | NA |
| Vegetable | Plantain Slices | 1 | 0 | 205 | Used Yucca Fries |
| Vegetable | Potato Wedges, Seasoned, Ketchup | 0 | 54 | NA | NA |
| Vegetable | Red Potatoes, Roasted | 1 | 0 | 56 | Combined Potato Wedges and Roasted Potatoes |
| Vegetable | Romaine, Raw | 0 | 1292 | NA | NA |
| Vegetable | Side Salad, Citrus Spinach | 1 | 4 | 283 | Combined Side Salad and Spinach Salad |
| Vegetable | Side Salad, Cucumber and Tomato | 1 | 11 | 290 | Combined Tomato and Cucumber Salad and Side Salad |
| Vegetable | Side Salad, Garden Side | 0 | 279 | NA | NA |
| Vegetable | Side Salad, Kale and Apple | 1 | 0 | 279 | Used Side Salad |
| Vegetable | Spinach, Cooked | 1 | 0 | 20th | 20th percentile vegetable |
| Vegetable | Sweet Potato Wedges | 0 | 458 | NA | NA |
| Vegetable | Turnips, Roasted | 1 | 0 | 20th | 20th percentile vegetable |
| Vegetable | Yuca, Baked | 1 | 0 | 205 | Used Yucca Fries |
| Vegetable | Yucca Fries, Ketchup | 0 | 205 | NA | NA |
| Vegetarian | Bean Burrito | 0 | 25 | NA | NA |
| Vegetarian | Bean Taco | 1 | 0 | 188 | Combined Black Beans and Black Beans and Corn |
| Vegetarian | Broccoli and Cheddar Croissant | 1 | 12 | 64 | Combined Broccoli and Cheddar Croissant and Cheese Sandwich |
| Vegetarian | Cheese Bites | 0 | 20 | NA | NA |
| Vegetarian | Cheese Pizza | 0 | 1781 | NA | NA |
| Vegetarian | Cheese Quesadilla | 1 | 9 | 233 | Combined Cheese Quesadilla and Chicken Quesadilla |
| Vegetarian | Grilled Cheese | 0 | 52 | NA | NA |
| Vegetarian | Hummus, Vegetables, Cheese, and Flatbread | 1 | 18 | 20th | 20th |
| Vegetarian | Macaroni and Cheese | 0 | 164 | NA | NA |
| Vegetarian | Marinated Tofu Banh Mi | 1 | 0 | 20th | 20th percentile vegetarian |
| Vegetarian | Marinated Tofu with Brown Rice | 1 | 0 | 20th | 20th percentile vegetarian |
| Vegetarian | Marinated Tofu with Cornbread | 1 | 0 | 20th | 20th percentile vegetarian |
| Vegetarian | Marinated Tofu with Lo Mein Noodles | 1 | 0 | 20th | 20th percentile vegetarian |
| Vegetarian | Marinated Tofu with Vegetable Fried Rice | 1 | 0 | 20th | 20th percentile vegetarian |
| Vegetarian | Pasta with Pesto | 1 | 0 | 32 | Used Pasta with Sauce |
| Vegetarian | Peanut Butter and Jelly Sandwich | 0 | 59 | NA | NA |
| Vegetarian | Spaghetti with Marinara and Cheese | 0 | 32 | NA | NA |
| Vegetarian | Spinach Quesadilla | 1 | 0 | 233 | Combined Cheese Quesadilla and Chicken Quesadilla |
| Vegetarian | Three Bean Chili with Cornbread | 1 | 0 | 131 | Used Three Bean Salad |
| Vegetarian | Three Bean Chili with Tortilla Chips | 1 | 0 | 131 | Used Three Bean Salad |
| Vegetarian | Toasted Cheese Sandwich and Three Bean Chili | 1 | 0 | 53 | Used Cheese Sandwich |
| Vegetarian | Vegetable Curry with Brown Rice | 1 | 0 | 80 | Used Chicken Curry with Brown Rice |
| Vegetarian | Veggie Pizza | 1 | 15 | 1360 | Combined Veggie Pizza and Pizza not specified |
| Vegetarian | White Garlic Pizza | 1 | 0 | 1781 | Combined Cheese Pizza and Pizza not specified |
| Vegetarian | Yogurt Grab and Go | 1 | 0 | 20th | 20th percentile vegetarian |

# SI Table 3. Weekly menus selected by optimization models.

| Model | Weekday | **Entrée** | | **Fruit** | **Vegetable** | **Milk** |
| --- | --- | --- | --- | --- | --- | --- |
|  |  | **Sub-Cat** | **Item** |  |  |  |
| MAX-CALCON | |  |  |  |  |  |
|  | Monday | Beef | Meatball Pizza | Applesauce | Baked Potato and Cauliflower, Roasted | Skim Chocolate Milk |
|  | Tuesday | Chicken | Pulled BBQ Chicken Sandwich | Mandarin Oranges, Canned | Beans, Baked Beans | Skim Chocolate Milk |
|  | Wednesday | Vegetarian | Peanut Butter and Jelly Sandwich | Mandarin Oranges, Canned | Cherry Tomatoes, Ranch and Romaine, Raw | Skim Plain Milk |
|  | Thursday | Chicken | Chicken Tenders with Dinner Roll | Watermelon, Fresh | Baked Potato and Cherry Tomatoes, Ranch | Skim Plain Milk |
|  | Friday | Beef | Hot Dog on a Bun with Popcorn | Watermelon, Fresh | Plantain Slices and Red Potatoes, Roasted | Skim Plain Milk |
|  |  |  |  |  |  |  |
| MAX-COSTCON | |  |  |  |  |  |
|  | Monday | Beef | Hamburger | Blueberries, Frozen | Beans, Baked Beans and Red Potatoes, Roasted | Skim Plain Milk |
|  | Tuesday | Chicken | Chicken Tenders with Dinner Roll | Mandarin Oranges, Canned | Cherry Tomatoes, Ranch | Skim Plain Milk |
|  | Wednesday | Vegetarian | Veggie Pizza | Watermelon, Fresh | Cucumber Slices, Ranch and Red Potatoes, Roasted | Skim Plain Milk |
|  | Thursday | Chicken | Pulled BBQ Chicken with Cornbread | Blueberries, Frozen | Plantain Slices | Skim Plain Milk |
|  | Friday | Beef | Hot Dog on a Bun with Popcorn | Mandarin Oranges, Canned | Romaine, Raw and Cherry Tomatoes, Ranch | Skim Plain Milk |
|  |  |  |  |  |  |  |
| MIN-COST | |  |  |  |  |  |
|  | Monday | Beef | Hamburger | Banana, Fresh | Beans, Sizzlin' Black Beans | 1% Plain Milk |
|  | Tuesday | Vegetarian | Three Bean Chili with Tortilla Chips | Raisins | x2 Carrots, Roasted | 1% Plain Milk |
|  | Wednesday | Chicken | Seasoned Chicken and Brown Rice | Watermelon, Fresh | Corn, Sweet Kernels and Peas | Skim Plain Milk |
|  | Thursday | Vegetarian | Grilled Cheese | Banana, Fresh | x2 Green Beans, Steamed | Skim Plain Milk |
|  | Friday | Beef | Steak and Cheese Croissant | Watermelon, Fresh | Romaine, Raw | Skim Plain Milk |
|  |  |  |  |  |  |  |

x2 = two servings of the vegetable

# SI Table 3 Cont. Weekly menus selected by optimization models.

| Model | Weekday | **Entrée** | | **Fruit** | **Vegetable** | **Milk** |
| --- | --- | --- | --- | --- | --- | --- |
|  |  | **Sub-Cat** | **Item** |  |  |  |
| MIN-DEV | |  |  |  |  |  |
|  | Monday | Chicken | Curry Chicken with Flatbread | Apple, Fresh | Beans, Chickpea Salad | Skim Plain Milk |
|  | Tuesday | Chicken | Chicken Tenders with Dinner Roll | Banana, Fresh | Romaine, Raw and Sweet Potato Wedges | Skim Plain Milk |
|  | Wednesday | Vegetarian | Cheese Bites | Blueberries, Frozen | Red Potatoes, Roasted and Green Beans, Steamed | Skim Plain Milk |
|  | Thursday | Vegetarian | Marinated Tofu with Vegetable Fried Rice | Banana, Fresh | Side Salad, Kale and Apple | Skim Plain Milk |
|  | Friday | Fish | Tuna with Pita Bread | Blueberries, Frozen | Romaine, Raw and Cherry Tomatoes, Ranch | Skim Plain Milk |
|  |  |  |  |  |  |  |
| MIN-DEV10 | | | |  |  |  |
|  | Monday | Chicken | Crispy Chicken Sandwich | Applesauce | Plantain Slices and Side Salad, Kale and Apple | Skim Plain Milk |
|  | Tuesday | Chicken | Chicken Tenders with Dinner Roll | Banana, Fresh | Romaine, Raw and Cherry Tomatoes, Ranch | Skim Plain Milk |
|  | Wednesday | Vegetarian | Cheese Bites | Blueberries, Frozen | Green Beans, Steamed | Skim Plain Milk |
|  | Thursday | Vegetarian | Marinated Tofu with Brown Rice | Banana, Fresh | Plantain Slices and Side Salad, Kale and Apple | Skim Plain Milk |
|  | Friday | Fish | Tuna with Pita Bread | Blueberries, Frozen | Beans, Chickpea Salad and Cherry Tomatoes, Ranch | Skim Plain Milk |
|  |  |  |  |  |  |  |
| MIN-DEV30 | | | |  |  |  |
|  | Monday | Vegetarian | White Garlic Pizza | Banana, Fresh | Romaine, Raw and Cherry Tomatoes, Ranch | Skim Plain Milk |
|  | Tuesday | Chicken | Chicken Tenders with Dinner Roll | Blueberries, Frozen | Beans, Sizzlin' Black Beans and Peppers, Cooked | Skim Plain Milk |
|  | Wednesday | Chicken | Crispy Chicken Sandwich | Watermelon, Fresh | x2 Plantain Slices and Cherry Tomatoes, Ranch | Skim Plain Milk |
|  | Thursday | Vegetarian | Marinated Tofu with Brown Rice | Banana, Fresh | x2 Red Potatoes, Roasted | Skim Plain Milk |
|  | Friday | Fish | Tuna with Pita Bread | Blueberries, Frozen | Baked Potato | Skim Plain Milk |
|  |  |  |  |  |  |  |
| MAX-HEI | |  |  |  |  |  |
|  | Monday | Chicken | Curry Chicken with Flatbread | Apple, Fresh | Beans, Chickpea Salad and Cherry Tomatoes, Ranch | Skim Plain Milk |
|  | Tuesday | Chicken | Crispy Chicken Sandwich | Banana, Fresh | x2 Broccoli, Roasted | Skim Plain Milk |
|  | Wednesday | Vegetarian | Cheese Bites | Pear, Fresh | Romaine, Raw and Broccoli, Steamed | Skim Plain Milk |
|  | Thursday | Vegetarian | Marinated Tofu with Vegetable Fried Rice | Apple, Fresh | Romaine, Raw and Cherry Tomatoes, Ranch | Skim Plain Milk |
|  | Friday | Fish | Baked Fish in Chip with Brown Rice | Pear, Fresh | Corn, Sweet Kernels and Green Beans, Steamed | Skim Plain Milk |

x2 = two servings of the vegetable

# SI Figure 1. Fruit frequency for baseline and optimized weekly menus.

# SI Figure 2. Vegetable frequency for baseline and optimized weekly menus.

# SI Table 4. Baseline and optimized weekly menus nutrient content, as offered and consumed.

|  | Energy (kcal) | | Fiber (g) | | Calcium (mg) | | Iron (mg) | |
| --- | --- | --- | --- | --- | --- | --- | --- | --- |
|  | Offered | Consumed | Offered | Consumed | Offered | Consumed | Offered | Consumed |
| Baseline | 3039 | 1744 | 44 | 21 | 2798 | 1704 | 20 | 12 |
| MAX-CALCON | 3247 | 2436 | 38 | 28 | 2494 | 1752 | 26 | 19 |
| MIN-COST | 2829 | 1544 | 49 | 21 | 3105 | 1809 | 19 | 9 |
| MAX-COSTCON | 2918 | 2193 | 34 | 25 | 2184 | 1486 | 20 | 15 |
| MAX-HEI | 2754 | 1381 | 58 | 23 | 2062 | 1242 | 16 | 7 |
| MIN-DEV | 2774 | 1508 | 52 | 25 | 1913 | 1175 | 14 | 7 |
| MIN-DEV10 | 3248 | 2094 | 58 | 32 | 1991 | 1257 | 15 | 9 |
| MIN-DEV30 | 3248 | 2218 | 52 | 32 | 2287 | 1535 | 20 | 14 |

# SI Table 4 Cont. Baseline and optimized weekly menus nutrient content, as offered and consumed.

|  | Polyunsaturated and Monounsaturated Fats (g) | | Added Sugar (g) | | Saturated Fat (g) | | Sodium (g) | | HEI | |
| --- | --- | --- | --- | --- | --- | --- | --- | --- | --- | --- |
|  | Offered | Consumed | Offered | Consumed | Offered | Consumed | Offered | Consumed | Offered | Consumed |
| Baseline | 54 | 31 | 13 | 8 | 31 | 19 | 4.749 | 2.756 | 84.18 | 76.86 |
| MAX-CALCON | 58 | 44 | 15 | 11 | 23 | 19 | 5.002 | 3.737 | 91.84 | 86.39 |
| MIN-COST | 42 | 23 | 5 | 2 | 30 | 18 | 4.767 | 2.409 | 80.92 | 78.62 |
| MAX-COSTCON | 52 | 40 | 6 | 5 | 21 | 17 | 4.071 | 3.064 | 91.72 | 86.90 |
| MAX-HEI | 47 | 24 | 1 | 1 | 7 | 4 | 3.520 | 1.734 | 98.02 | 93.27 |
| MIN-DEV | 58 | 32 | 1 | 1 | 10 | 5 | 3.857 | 1.961 | 96.77 | 94.47 |
| MIN-DEV10 | 71 | 45 | 1 | 1 | 10 | 7 | 3.575 | 2.091 | 99.80 | 92.45 |
| MIN-DEV30 | 54 | 38 | 1 | 1 | 15 | 12 | 4.621 | 2.916 | 96.23 | 91.33 |
